# Adaptive sequence evolution is driven by biotic stress in a pair of orchid species (*Dactylorhiza*) with distinct ecological optima

**DOI:** 10.1111/mec.14123

**Published:** 2017-04-27

**Authors:** Francisco Balao, Emiliano Trucchi, Thomas M. Wolfe, Bao‐Hai Hao, Maria Teresa Lorenzo, Juliane Baar, Laura Sedman, Carolin Kosiol, Fabian Amman, Mark W. Chase, Mikael Hedrén, Ovidiu Paun

**Affiliations:** ^1^ Department of Botany and Biodiversity Research University of Vienna Vienna Austria; ^2^ Departamento de Biología Vegetal y Ecología University of Seville Sevilla Spain; ^3^ Department of Life Sciences and Biotechnologies University of Ferrara Ferrara Italy; ^4^ Vienna Graduate School of Population Genetics Vienna Austria; ^5^ Gregor Mendel Institute for Plant Molecular Biology Vienna Austria; ^6^ Institut für Populationsgenetik Vetmeduni Vienna Vienna Austria; ^7^ Centre of Biological Diversity School of Biology University of St Andrews St Andrews UK; ^8^ Department of Chromosome Biology University of Vienna Vienna Austria; ^9^ Royal Botanic Gardens Kew Richmond UK; ^10^ School of Plant Biology University of Western Australia Crawley, Perth WA Australia; ^11^ Department of Biology Lund University Lund Sweden

**Keywords:** abiotic stress, defence, ecological divergence, positive selection, small RNAs, transcriptomics

## Abstract

The orchid family is the largest in the angiosperms, but little is known about the molecular basis of the significant variation they exhibit. We investigate here the transcriptomic divergence between two European terrestrial orchids, *Dactylorhiza incarnata* and *Dactylorhiza fuchsii*, and integrate these results in the context of their distinct ecologies that we also document. Clear signals of lineage‐specific adaptive evolution of protein‐coding sequences are identified, notably targeting elements of biotic defence, including both physical and chemical adaptations in the context of divergent pools of pathogens and herbivores. In turn, a substantial regulatory divergence between the two species appears linked to adaptation/acclimation to abiotic conditions. Several of the pathways affected by differential expression are also targeted by deviating post‐transcriptional regulation via sRNAs. Finally, *D. incarnata* appears to suffer from insufficient sRNA control over the activity of RNA‐dependent DNA polymerase, resulting in increased activity of class I transposable elements and, over time, in larger genome size than that of *D. fuchsii*. The extensive molecular divergence between the two species suggests significant genomic and transcriptomic shock in their hybrids and offers insights into the difficulty of coexistence at the homoploid level. Altogether, biological response to selection, accumulated during the history of these orchids, appears governed by their microenvironmental context, in which biotic and abiotic pressures act synergistically to shape transcriptome structure, expression and regulation.

## 1 Introduction

The orchid family represents an extraordinary biological diversification, containing over 8% of all flowering plant species, making Orchidaceae the largest family of angiosperms (Chase et al., 2015; Givnish et al., 2015). They are distributed from equatorial lowlands to arctic and alpine locations, through a remarkable variability, affecting their morphology, ecology, genomes and life history strategies (Benzing, 1986). Orchids are particularly renowned for their specialized and diverse pollination systems (Darwin, 1877), and pollinator‐driven isolation is often discussed as a main factor driving their diversification (Benzing, 1986; Cozzolino & Widmer, 2005). However, a third of all orchids are estimated to be pollinated on a deceptive basis (i.e., they offer no reward, Tremblay, Ackerman, Zimmerman, & Calvo, 2005), often with the help of unspecific pollinators. Indeed, pollinator‐mediated reproductive isolation has been shown to play little or no role in food‐deceptive orchids (Moccia, Widmer, & Cozzolino, 2007).

Other factors put forward to explain orchid diversity range from epiphytism (Gravendeel, Smithson, Slik, & Schuiteman, 2004; Silvera, Santiago, Cushman, & Winter, 2009), low fruiting success setting the stage for a dynamic interplay between drift and natural selection (Tremblay et al., 2005), obligate orchid–mycorrhiza interactions (Tupac Otero & Flanagan, 2006), CAM photosynthesis and tropical distribution (Silvera et al., 2009) or a combination of these factors (Givnish et al., 2015). In general, orchid species diversity has been reported to correlate to area size and, even more strongly, with habitat heterogeneity (e.g., with maximum island elevation as a surrogate for habitat diversity in the Caribbean, Ackerman, Trejo‐Torres, & Crespo‐Chuy, 2007), indicating that ecological conditions may be a major driver of diversity in the family. However, only little is known about the molecular factors underlying divergence between orchid species, mainly due to disproportionately limited genomic data in public databases despite the recent availability of a reference genome for *Phalaenopsis equestris* (Cai et al., 2015).

The molecular basis of biodiversity can reside in both amino acid sequence variation (Nielsen, 2005) and regulatory divergence that triggers protein abundance shifts (Wray, 2007), and these molecular components may evolve at different rates. For example, accumulating evidence suggests a role for divergent small RNAs (sRNAs) and other regulatory elements in postzygotic reproductive isolation, and the hybrid incompatibility they trigger is predicted to evolve relatively early in the speciation process (Ha, Pang, Agarwal, & Chen, 2008; Hollister et al., 2011; Kitano, Yoshida, & Suzuki, 2013; McManus et al., 2010; Michalak, 2008). Small RNAs, including plant microRNAs (miRNAs), secondary small interfering RNAs (siRNAs) and heterochromatic small interfering RNAs (hetsiRNAs, Borges & Martienssen, 2015), are involved in (post)transcriptional gene regulation, RNA‐directed DNA methylation and chromatin remodelling, playing major roles in the maintenance of genome stability and function (Zhang, 2008). Importantly, regulatory divergence is generally dynamic, potentially exposing phenotypes to natural selection differentially across time and environmental conditions. Biological responses to selection depend heavily on the environmental context, with regard to biotic and abiotic conditions that can act individually or synergistically to either accelerate or constrain trait evolution (Anderson, Wagner, Rushworth, Prasad, & Mitchell‐Olds, 2014). Understanding the extent and timescale of the interactions between different molecular levels in response to the plethora of characteristics of complex environments is a primary goal of evolutionary biology.

In this study, we aimed to investigate the molecular basis of adaptation to specific microenvironments in *Dactylorhiza fuchsii* and *D. incarnata*, two terrestrial, food‐deceptive orchid species (Sletvold, Grindeland, & Ågren, 2010) that provide a suitable model to trace effects of natural selection. The two orchid species broadly share geographical distribution (northern and central Europe and Western Asia, Tutin et al., 1980) and often grow in proximity. However, they have clearly distinct morphologies and also differ in their ecological preference (Paun et al., 2011). *Dactylorhiza fuchsii* inhabits grasslands, open woods and disturbed habitats such as road margins, whereas *D. incarnata* prefers marshes, fens and bog habitats. The genetic structure of the two species has most likely been shaped by different histories as, in contrast to *D. fuchsii*,* D. incarnata* appears to have experienced a dramatic reduction in population size that homogenized its genetic variation at neutral markers (Balao, Tannhäuser, Lorenzo, Hedren, & Paun, 2016; Hedrén, 1996; Pillon et al., 2007), despite its current wide distribution and its significant morphological heterogeneity. The two species have hybridized on multiple occasions, producing an array of ecologically divergent allotetraploid sibling species (Devos, Raspe, Oh, Tyteca, & Jacquemart, 2006; Hedrén, 1996; Hedrén, Fay, & Chase, 2001; Pillon et al., 2007), several of which are also broadly distributed in Europe.

With RNAseq across several replicates originating from different European regions, we searched for genes with lineage‐specific evolutionary patterns shaped by positive selection, and tested for differential expression (DE) that correlate to phenotypic divergence between the two species (De Wit, Pespeni, & Palumbi, 2015). We further aimed to time the divergence between the two species and tested whether the coding single nucleotide polymorphism (cSNP) patterns obtained follow the low neutral genetic diversity in *D. incarnata*. Finally, we sequenced sRNAs to track divergent post‐transcriptional gene regulation (Ehrenreich & Purugganan, 2008). One specific aim within the latter analysis was to search for a potential deviating negative control of transposable elements (TEs) that may have resulted in the difference in genome size between the two species (1C values: 3.55 pg for *D. incarnata* vs. 2.98 pg for *D. fuchsii*, Aagaard, Sastad, Greilhuber, & Moen, 2005). Besides understanding in detail the molecular divergence between the diploid orchid species, this work is also setting a foundation for further studies of the molecular principles underlying widespread allopolyploidy in this orchid group and its impact on evolutionary novelty and ecological diversification.

## 2 Materials and Methods

### 2.1 RNAseq and de novo transcriptome assemblies

We sequenced leaf RNAseq libraries (see Appendix S1) of five wild individuals each of *D. fuchsii* and *D. incarnata* from different European regions (Table 1, Fig. S1) after they had been transplanted and grown in a common garden in Vienna, Austria, for at least one growth cycle in order to remove temporary environmental effects. Every year, *Dactylorhiza* plants produce a new tuber that carries them through to the next year. We focused on leaves as an ecologically relevant tissue, which also avoids potential contamination by mycorrhizal fungi associated with orchid roots. Across the individuals analysed, the directional RNA sequencing generated almost 1.5 billion pairs of 100‐bp reads (Table 1).

**Table 1:** Details of the leaf samples analysed in this study

Region	No.	Acronym	Collectors	Latitude/Longitude	Raw pairs of RNAseq reads (M)	Raw sRNAseq reads (M)
*D. fuchsii*						
Pannonian	1566	fA6	GS, OP	47°53′41″N 19°52′01″E	226.8	16.7
Pyrenees	1001	fP1	MC, MF, OP	43°12′43″N 0°49′50″E	79.4	23.8
Pyrenees	1707	fP7	OP	42°53′40″N 1°55′46″E	166.0^a^	21.8
Britain	1804	fB4	FB, OP	54°15′10″N 0°41′06″W	107.1	18.6
Britain	1855	fB5	FB, OP	57°40′03″N 7°14′32″W	224.3	10.4
*D. incarnata*						
Alps	1586	iA6	OP	47°16′54″N 11°11′04″E	199.8	5.3
Scandinavia	1904	iS4	MH	57°20′26″N 18°19′16″E	82.0	13.1
Scandinavia	1908	iS8	MH	57°49′01″N 18°53′43″E	201.1	8.9
Britain	1176	iB6	RB	57°40′19″N 7°14′17″W	101.0	‐
Britain	1870	iB0	FB, OP	57°40′14″N 7°14′22″W	75.4	7.7

Collector abbreviations are FB, Francisco Balao; GS, Gabor Szramko; MC, Mark Chase; MF, Michael Fay; MH, Mikael Hedrén; OP, Ovidiu Paun and RB, Richard Bateman.

a Summed over two half lanes.

As the closest assembled genome (*P. equestris*, Cai et al., 2015) is highly divergent from *Dactylorhiza* (their most recent common ancestor lived 55–70 Ma, Givnish et al., 2015), we have assembled de novo with *Trinity* (Haas et al., 2013) a combined reference transcriptome for both *Dactylorhiza* species (for details see Appendix S1 and Fig. S2). The individual transcriptomes are available from GenBank Transcriptome Shotgun Assembly (TSA) Database (Accession nos.: GFHX00000000 and GFJV00000000 for *D. fuchsii* and *D. incarnata*, respectively). The combined final transcriptome is available from https://plantgenomics.univie.ac.at/downloads/. Due to its low number of assembled contigs pointing to a technical depletion of complexity, and due to its outlier position in ordination analyses, the individual fA6 was excluded from all further RNAseq analyses.

### 2.2 Variant discovery and assessment of genetic diversity

In order to conduct evolutionary analyses, the filtered RNAseq reads were mapped to an available, closely related outgroup reference, the transcriptome of *Orchis italica* (De Paolo, Salvemini, Gaudio, & Aceto, 2014), which is a member of the same orchid subtribe (Chase et al., 2015). The mapping has been performed with the second‐pass approach (Engström et al., 2013) of star v2.4.1d (Dobin et al., 2013), by lowering the maximum allowed ratio of mismatches to read length to 0.11. The best practices recommendations (DePristo et al., 2011; Van Auwera et al., 2013) for gatk version 3 (McKenna et al., 2010) have been followed, but with a hybrid approach between RNA and DNA sequencing as the analyses have been performed on a reference transcriptome, not a full genome. After processing the BAM files by adding read groups and removing duplicates with picard tools (v.1.119, http://broadinstitute.github.io/picard/), we split the reads into exon segments and reassigned star mapping qualities. The indelrealigner module from gatk v3.5‐0‐g36282e4 was used to improve local alignments around indels, downsampling to a maximum of 100× coverage. Variants were further called for each sample in the GVCF mode of the gatk haplotypecaller to generate an intermediate gVCF, by ignoring any soft clipped bases in the reads to minimize false positives and negative calls. Next, we processed all samples in the cohort in a joint genotyping analysis with genotypegvcfs with the minimum Phred‐scaled confidence threshold of 20 at which variants should be called and emitted, following gatk best practices recommendations for RNAseq. After retaining only SNPs with the selectvariants module of gatk to avoid later uncertainties in alignments, variants have been further filtered out if any of the following criteria were fulfilled: the quality normalized by the coverage (QD) was <2.0, the Phred‐scaled *p*‐value for Fisher's exact test to detect strand bias (FS) was >60.0, or the root mean square of mapping quality across all samples (MQ) was <40. Diversity measures were calculated with vcftools v.0.1.14 (Danecek et al., 2011) with the options –het (for the per‐individual heterozygosity and inbreeding coefficient *F*), –site‐pi (for per site nucleotide divergence π) and –singletons (for private alleles).

For visualizing genetic relationships between individuals, we filtered out with vcftools the variants with data in less than six (of the nine) *Dactylorhiza* individuals. The 449,518 retained SNPs were further concatenated to a Phylip file with pgdspider v.2.0.9.2 (Lischer & Excoffier, 2012), summarizing heterozygosities as IUPAC ambiguities. A maximum‐likelihood phylogenetic tree with 1,000 rapid bootstrap inferences and a GTRCAT substitution model were inferred with raxml v.7.2.8 (Stamatakis, 2014) and the results were graphically visualized with figtree v.1.4.2 (available from http://tree.bio.ed.ac.uk/software/figtree/).

The genetic similarity among individuals of the two *Dactylorhiza* species was also visualized with a principal component analysis (PCA). The data were first filtered with vcftools by retaining only SNPs with the minor allele present in at least two individuals. The retained 129,511 biallelic SNPs were imported in the bioconductor (Huber et al., 2015) with gdsfmt, and the PCA was finally drawn with the r package snprelate (Zheng et al., 2012).

### 2.3 Rates of sequence evolution and targets of selection

Synthetic transcripts of each sample have been generated from the gatk‐filtered vcf file with the gatk fastaalternatereferencemaker, using IUPAC encodings for heterozygote states. We further used for each sample the gatk callableloci with default settings to filter only the callable transcriptomic intervals. These have been intersected with bedtools v.2.25.0 (Quinlan & Hall, 2010) with exonic regions of the *O. italica* reference predicted with transdecoder (v.2.0.1, https://transdecoder.github.io/). bedtools getfasta has been used to retain from each individual only the target intervals. Finally, indel‐free coding DNA sequence (CDS) alignments of at least 150‐bp coding regions have been retained for positive selection analyses.

Nonsynonymous (*K*_a_) and synonymous (*K*_s_) substitution rates for CDS alignments of pairwise combinations of sequences of *D. fuchsii*,* D. incarnata* and *O. italica* were first calculated using an approximate method (MYN) modified after Yang and Nielsen (2000) and implemented in the software package kaks_calculator v.2.0 (Zhang, Li, & Yu, 2006). The level of significance of Fisher's exact tests was adjusted with the false discovery rate (FDR) approach with the p.adjust function in r. The contigs shown to be under positive selection (i.e., *K*_a_/*K*_s_ > 1) between *D. incarnata* and *D. fuchsii* within an FDR level <0.1 have been confirmed by estimating pairwise dN/dS (ω) with a maximum‐likelihood method implemented in codeml of the paml package v.4.8a (Danecek et al., 2011). To test for statistical significance (*p *<* *.05), a likelihood ratio test was applied against a model with fixed ω = 1 (Wong, Yang, Goldman, & Nielsen, 2004). We further aimed to detect the particular branch on which positive selection acted by performing branch‐site model tests M1a versus M2a (Zhang, Nielsen, & Yang, 2005) in codeml, comparing models with estimated ɷ versus the corresponding null models with fixed ω = 1 along the *D. fuchsii* branch, and the *D. incarnata* branch, respectively. A simple phylogenetic tree has been used for the inference as ((*D. fuchsii*,* D. incarnata*), *O. italica*)). Using the initial list of tested contigs as reference set and the genes under positive selection on each branch as the test sets, GO term enrichments were determined with Fisher's exact tests and a *p*‐value <.01 in blast2go to place the genes in a meaningful context. The GO terms were summarized with revigo as explained above.

### 2.4 Abundance estimation

For analyses of DE, the filtered RNAseq reads were mapped to the *Dactylorhiza* reference using star v.2.4.1b (Dobin et al., 2013) with default settings allowing for up to 20 multiple mapping reads. A unigene‐level table of counts was obtained with corset v.1.04 (Davidson & Oshlack, 2014), by filtering out any transcripts with fewer than 18 reads aligning over the nine samples. corset uses multimapping reads to hierarchically cluster similar transcripts based on their expression patterns and proportion of shared reads. Three different methods within bioconductor were used for DE analyses, deseq2 v. 1.6.3 (Anders & Huber, 2010), bayseq v. 2.0.50 (Hardcastle & Kelly, 2010) and edger v. 3.8.6 (Robinson, McCarthy, & Smyth, 2010). Clusters were considered DE if they were detected in each of the three methods used at an FDR <0.05, in order to reduce the rate of false positives. Using a custom *python* script, the longest transcript from each corset cluster was extracted and used for further GO enrichment analyses in blast2go v.3.2.7 (Conesa & Götz, 2008). Fisher's exact tests were implemented at a threshold *p*‐value of .01, using as the reference set the list of the longest contigs for each cluster in the data. Overrepresented GO terms were then summarized using revigo (http://revigo.irb.hr, Supek, Bošnjak, Škunca, & Šmuc, 2011), applying thinning based on semantic similarity with the *SimRel* algorithm (Schlicker, Domingues, Rahnenführer, & Lengauer, 2006).

### 2.5 Small RNA analysis

For the same *Dactylorhiza* individuals used in the RNA sequencing experiment (i.e., including individual fA6, but excluding iB6, for which not enough leaf material was still available at this stage), we also sequenced leaf sRNAseq libraries as multiplexed 50‐bp single‐end Illumina libraries (see Appendix S1 and Table 1). After demultiplexing and adapter removal, we selected for each accession the reads with lengths from 20 to 22 nucleotides (hereafter “mi/tasiRNA”) and, separately, of 24 nucleotides (hereafter “siRNA”) and analysed them as two classes. Length‐selected sRNA reads were mapped to the *Dactylorhiza* reference transcriptome using star v2.5.1b (Dobin et al., 2013) not allowing for any mismatch (–outFilterMismatchNmax 0) and discarding any read mapping to more than one contig (–outFilterMultimapNmax 0). These settings for mapping on the *Dactylorhiza* reference transcriptome were chosen to include only unambiguous signals in further analyses. A replicated analysis allowing for one mismatch produced consistent results. Read counts per contig were further summarized in corset with default settings and the resulting tables of counts were then imported into *R*. Those contigs showing less than 10 reads in at least three individuals in one of the two species were filtered out. The function *betweenLaneNormalization* in the r package edaseq (bioconductor) was used for sequencing depth normalization among samples applying a nonlinear full quantile method. A further normalization step based on a factor analysis of putative nondifferentially regulated genes was performed. First, a set of nondifferentiated empirical control genes was produced in silico by running a preliminary analysis in edger (Robinson et al., 2010) and selecting all genes scored as undifferentiated (FDR > 0.95). This list of empirical genes was then used as negative control in the *RUVg* normalizing function in the r package ruvseq (Risso, Ngai, Speed, & Dudoit, 2014) removing *k* = 2 factors of unwanted variation (*k *=* *1 or 3 were also tested). Results of the applied normalization were checked by plotting the relative log expression (*plotRLE* function) and the PCA (*plotPCA* function) across samples using the r package edaseq. The model resulting from the *RUVg* analysis was then used in edger, together with the group assignation, to implement the design of the differential regulation analysis between *D. fuchsii* and *D. incarnata*. The *glmLRT* function was used in edger and a FDR <0.05 was applied as a threshold for identifying differentially regulated transcripts. Differentially overregulated contigs were used in GO enrichment analyses in blast2go. All transcriptome contigs were set as a reference in Fisher's exact tests and a *p*‐value of .01 was applied as a threshold. Overrepresented GOs were then visualized in *REVIGO* as explained above.

### 2.6 Assessment of ecological divergence

We finally aimed to characterize interspecific ecological divergence and place our transcriptomic results in this context. Locality information was collected from *Global Biodiversity Information Facility* (GBIF) using the r package rgbif (available from https://cran.r-project.org/web/packages/rgbif). For robustness, we restricted the data set to localities with known herbarium vouchers. A few additional localities from our personal observations were also added. The geographical information was converted into decimal degrees and cleaned by removing exact duplicate localities. Locally dense sampling was finally reduced by thinning the records to one per 10‐km^2^ grid cell. Based on occurrence information at 298 localities for *D. fuchsii* and 393 for *D. incarnata*, spatial environmental data were extracted with raster (available from https://cran.r-project.org/web/packages/raster) from Bioclim (www.worldclim.org/bioclim) and Landsat Tree Cover (http://glcf.umd.edu/data/landsatTreecover/). We removed highly correlated bioclimatic variables in the obtained data set using a threshold of 0.80 using the package caret (available from https://cran.r-project.org/web/packages/caret). This retained only seven of the 19 Bioclim variables for further analyses: annual mean temperature (BIO1), mean diurnal temperature range (BIO2), isothermality (BIO3), mean temperature of wettest quarter (BIO8), annual precipitation (BIO12), precipitation seasonality (BIO15) and precipitation of warmest quarter (BIO18). Ecological niche overlap between the two species was tested with ecospat v.1.1 (available from https://cran.r-project.org/web/packages/ecospat). This method calculates the kernel smoothing densities of species occurrences and climatic variables along environmental axes from a PCA. Then, niche overlap along these axes was assessed with three statistical tests: niche similarity/equivalency (Broennimann et al., 2012), Schoener's D metric (Schoener, 1970) and a niche divergence test (McCormack, Zellmer, & Knowles, 2010) based on the differences in PCA scores between species in comparison with the differences in scores for distinct “background regions” for each species. For these tests, we extracted climate data from 2,000 randomly selected points (background region). These points were randomly selected from polygons around the projected coordinate occurrences with a 10‐km buffer. For the null distribution of D statistics, we used 1,000 replicates and a grid environmental value of 100. For the differences in PCA scores in the niche divergence test, to calculate the 95% confidence interval, we used a resampling approach based on 1,000 replicates of 500 samples for each species’ background region.

Finally, soil pH at ca. 7 cm depth near 45 *D. incarnata* and 19 *D. fuchsii* plants has been measured with a Direct Soil pH Meter HI99121 (Hanna Instruments) across nine and, respectively, five populations from Britain, Scandinavia, Alps and Pyrenees between 2010 and 2015. The data have been summarized with box plots, and significance of the differences between the pH preferences of the two species has been tested with a *t* test in r.

## 3 Results

### 3.1 Variation analyses and rates of sequence evolution

**Figure 1:**
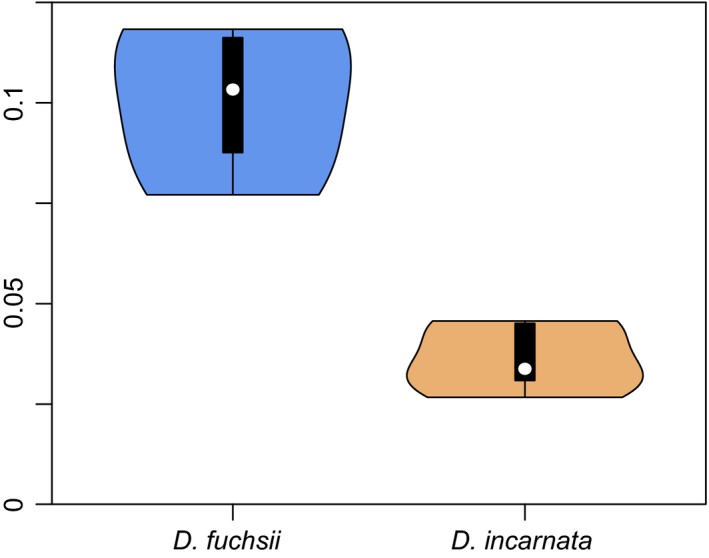
Violin plot of the proportions of heterozygous SNPs per individual among the 682,118 high‐quality, filtered variant positions [Colour figure can be viewed at wileyonlinelibrary.com]

Mapping efficiency on the *Orchis* reference transcriptome ranged from 77% (fP1) to 87% (fB5) in *D. fuchsii* and from 82% (iB0) to 87% (iA6) for *D. incarnata* individuals. Initially, the gatk (McKenna et al., 2010) variant calling pipeline identified 23,185 indels and 727,350 SNPs in the data. After retaining only SNPs and implementing a set of variant filters in gatk, we retained 682,118 high‐quality SNPs, of which 11,583 were multiallelic across the *Dactylorhiza–Orchis* group. The average transition/transversion rate found was 1.61. Heterozygosity was higher in *D. fuchsii* individuals than in *D. incarnata* (Figure 1), resulting in increased inbreeding coefficient *F* in *D. incarnata* (Table 2). Between individuals, diversity measures and the number of private alleles were also higher in *D. fuchsii* individuals than those in *D. incarnata* (Table 2). A maximum‐likelihood phylogenetic tree (Fig. S3) and a PCA based on cSNPs (Fig. S4a) separated clearly the two species. Less significant, but clear geographical patterns were also visible.

**Table 2:** Summary of cSNP diversity measures in the *Dactylorhiza* transcriptomes

	*H*_O_	*F*	*P*	π
fB4	0.12	0.73	0.40	
fB5	0.09	0.79	0.24	
fP1	0.08	0.81	0.16	
fP7	0.12	0.72	0.25	
*D. fuchsii*	**0.1**	**0.76**	**0.26**	**.077**
iA6	0.03	0.92	0.06	
iB6	0.05	0.89	0.19	
iB0	0.03	0.92	0.14	
iS4	0.03	0.94	0.11	
iS8	0.05	0.89	0.19	
*D. incarnata*	**0.04**	**0.91**	**0.14**	**.037**

*H*_O_, observed proportion of heterozygous SNPs; *F*, per‐individual inbreeding coefficient; *P*, ratio of private variants (i.e., singletons or doubletons) to total number of variant sites; π, nucleotide diversity per species averaged over all biallelic SNP positions with more than two genotypes present per group. Species values are given in bold.

**Figure 2:**
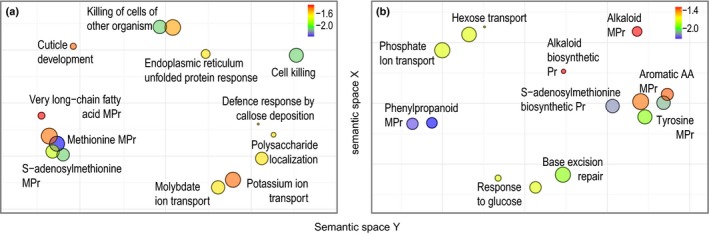
Enriched biological processes (*p *<* *.01) with elements targeted by positive selection in *D. fuchsii* (a) and in *D. incarnata* (b). Bubble size is proportional to the frequency of the respective term in the public GO database. The colour represents the log10 value of the significance of Fisher's exact tests of enrichment, corresponding to the indicated scale. Pr, process; MPr, metabolic process

The positive selection analyses included 5,082, 3,290 and 2,923 indel‐free pairwise CDS alignments longer than 150 bp for *D. fuchsii–O. italica*,* D. incarnata–O. italica* and *D. fuchsii–D. incarnata*, respectively (Table 3). The average values of *K*_s_ and *K*_a_, as indicated by kaks_calculator (Zhang et al., 2006) analyses, were similar for both *Dactylorhiza*–*Orchis* comparisons (i.e., *K*_s_ = 0.1 and, respectively, *K*_a_ = 0.02), whereas for the *D. fuchsii–D. incarnata* comparison lower values have been obtained as expected because of their more recent divergence (*K*_s_ = 0.062, *K*_a_ = 0.014; Fig. S5). Based on the *K*_s_ values and the divergence time of ca. 16.75 Ma previously estimated for the split *Dactylorhiza–Orchis* (Givnish et al., 2015; Inda, Pimentel, & Chase, 2012), the ancestral lineages of the two *Dactylorhiza* species split around 10.4 Ma. Between 61% and 67% of transcripts with alignments showed signs of purifying selection (i.e., *K*_a_/*K*_s_ < 1, FDR < 0.1; Table 3). For the pair *D. fuchsii–D. incarnata,* 41 putative CDS were inferred to be under positive selection by the approximate MYN method implemented in kaks_calculator (i.e., *K*_a_/*K*_s_ > 1, FDR < 0.1), of which 35 (Appendix S2) were confirmed (i.e., ω > 1, *p* < .05) with the maximum‐likelihood approach implemented in paml (Yang, 2007). Among them, codeml branch‐site model tests have indicated 18 CDS to be under positive selection on the *D. fuchsii* branch and 14 on the *D. incarnata* branch. Enrichment tests found for the *D. fuchsii* and *D. incarnata* branches 27 and 56 enriched GO terms, respectively (Appendix S2), related mostly to response to stress, primarily biotic, but also abiotic (Figure 2, Fig. S6).

**Table 3:** Summary of the kaks_calculator analysis of rates of sequence evolution in pairwise alignments over 150 bp of the *Dactylorhiza* species and *O. italica*

	Alignments	AvKs	AvKa	Purifying	Positive
*D. fuchsii–O. italica*	5,082	0.096	0.022	3,416	79
*D. incarnata–O. italica*	3,290	0.096	0.021	2,059	66
*D. fuchsii–D. incarnata*	2,923	0.062	0.014	1,790	41

AvKs, average synonymous substitution rate, AvKa, average nonsynonymous substitution rate, purifying and positive – the number of putative CDS showing *K*_a_/*K*_s_ <1 and, respectively, >1 at an FDR < 0.1.

### 3.2 Abundance estimation and DE analyses

**Figure 3:**
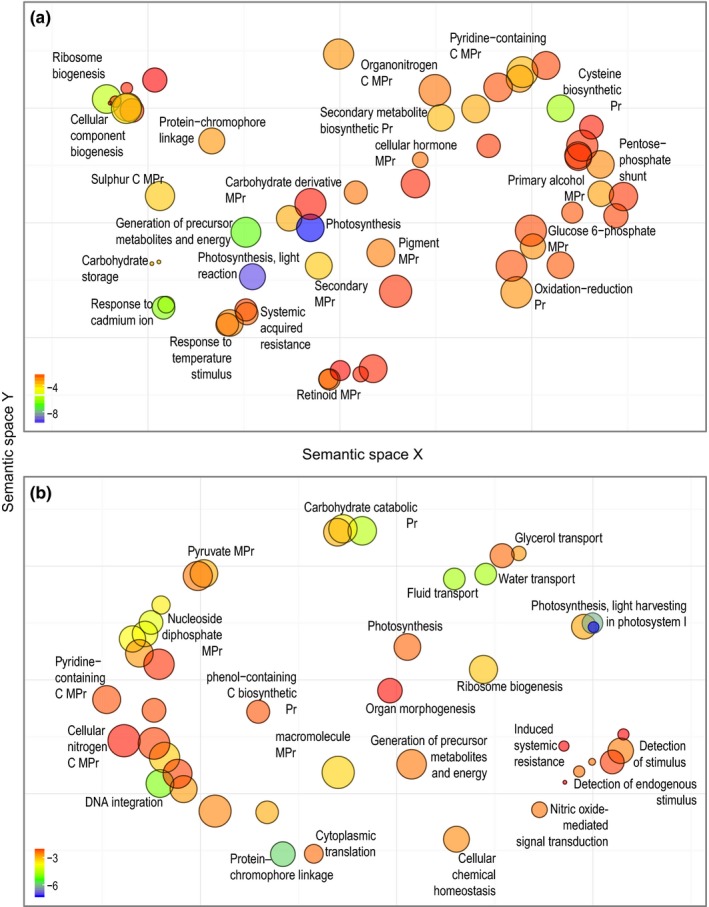
revigo visualization of the enriched biological processes (*p *<* *.01) that are affected by differential expression (FDR < 0.05) between *D. fuchsii* and *D. incarnata*. (a) Enriched GO terms of genes overexpressed in *D. fuchsii*. (b) Enriched GO terms of genes overexpressed in *D. incarnata*. Bubble size is proportional to the frequency of the respective term in the public GO database. The colour represents the log10 value of the significance of Fisher's exact tests of enrichment, corresponding to the indicated scale. Pr, process; C, compound; MPr, metabolic process

The table of counts included 41,851 retained clusters (i.e., unigenes). A PCA (Fig. S4b) drawn with edger confirmed that gene expression variation between the two *Dactylorhiza* species explained the largest principal component (70.5% of the total variation), but some geographical structure was also evident, in particular in *D. incarnata*. Reflecting the substantial interspecific expression variation, DE tests indicated a significant difference in expression between the two *Dactylorhiza* species (Fig. S7), ranging from 14,165 clusters (edger) to 14,866 clusters (bayseq). A total of 13,157 DE clusters were present in each of the three tests at an FDR < 0.05; of these, 47% were found to be overexpressed in *D. fuchsii*. Some of these genes have not yet been characterized, but Fisher's exact tests on annotated genes identified 184 enriched GO terms with overexpressed elements in *D. fuchsii* and 133 in *D. incarnata* (Appendix S3). The GO terms that had elements affected by DE were of a broad spectrum (Figure 3, Fig. S8): photosynthesis, chlorophyll and pigment binding, ribosome biogenesis, generation of precursor metabolites and energy, protein–chromophore linkage, structural molecule activity and tetrapyrrole binding were some of the significantly enriched GO terms in upregulation of both *D. fuchsii* and *D. incarnata*, but representing different transcripts. Enriched GO terms specifically overexpressed in *D. fuchsii* included, for example, response to temperature, carbohydrate storage, systemic acquired resistance, organonitrogen metabolism and cysteine biosynthetic process (Figure 3a, Fig. S8a). For *D. incarnata*, specific enriched overexpressed GO terms included a variety of fluid transmembrane transport‐related GO terms, light harvesting in photosystem I, carbohydrate catabolic processes, DNA integration, RNA‐dependent DNA polymerase activity, but also, perhaps importantly, the induced systemic resistance, detection of stimulus and nitric oxide signal transduction (Figure 3b, Fig. S4b).

### 3.3 Small RNA analysis

After selection by length, we retained an average of 3.1 million 20‐ to 22‐nt‐long reads per sample and an average of 1.5 million 24‐nt‐long reads per sample, respectively. Average mapping efficiency of these two categories of reads to the *Dactylorhiza* reference transcriptome was 30.1% (*SD* = 2.8%) and 21.3% (*SD* = 6.7%), respectively. This is in line with the expectation that an important part of sRNAs target genomic regions outside of the transcriptome (e.g., introns, repetitive elements and promoters), albeit some of the active repetitive elements and parts of introns can be represented in the reference transcriptome constructed from a total RNA library.

Clustering of the samples (Fig. S4c,d), based on the siRNAs and, respectively, the mi/tasiRNAs confirmed a significant divergence in regulation between the two species. The differential regulation tests resulted in 1,232 contigs overregulated by mi/tasiRNAs in *D. fuchsii* and 1,449 contigs in *D. incarnata* over a total of 9,848 transcripts passing initial filters. Enrichment tests identified 48 enriched GO terms with mi/tasiRNA overregulated elements in *D. fuchsii* and 38 in *D. incarnata*. DNA integration, DNA metabolism, nitrogen metabolism, RNA‐directed DNA polymerase (RdDp) activity, nucleic acid binding, heterocyclic and organic cyclic compound binding are some of the significantly enriched GO terms in the set of contigs overregulated in *D. fuchsii* (Fig. S9). In *D. incarnata*, several of the enriched GO terms were related to organelle function (e.g., light harvesting in photosystem I, protein–chromophore linkage, chlorophyll and pigment binding) and some to iron transmembrane transport. Concerning the siRNA, 6,001 contigs passed initial filters and 855 and 1,045 contigs were overregulated in *D. fuchsii* and *D. incarnata*, respectively. Fisher's exact tests gave evidence for 35 GO terms (Fig. S10) with elements overregulated by siRNAs in *D. fuchsii* and 30 in *D. incarnata*. In this case, the most significant GO terms enriched in both species appeared related to DNA integration, DNA metabolism, nucleic acid metabolism, binding, nucleic acid binding, zinc ion binding, cation binding and heterocyclic and organic cyclic compound binding (Fig. S10). Several GO terms specifically overregulated in *D. fuchsii* included metabolism and modifications of small nuclear (snRNA), small nucleolar RNAs (snoRNA) and pseudouridine synthesis.

### 3.4 Ecological differentiation

As Bioclim parameters have a resolution of 30 arc seconds (ca. 1 km^2^), they are considered representative for the macrohabitat preference of each species. A test of climatic niche overlap (Broennimann et al., 2012) indicated that overall, the (macro)habitats of the two species were similar (*D* = 0.75; similarity tests in both directions with *p* < .01), but an equivalency test (*p* = .002) indicated that the climatic niches of the two species were not completely identical. Accordingly, a divergence test of the climatic niches (McCormack et al., 2010) showed a significant differentiation on the PC2 niche axis (mean divergence = −0.36, null hypothesis = −0.21 to 0.13; paired samples Student's *t* = −3.650, *df* = 624.5, *p* < .001), summarizing annual mean temperature (BIO1), isothermality (BIO3), mean temperature of wettest quarter (BIO8), annual precipitation (BIO12) and precipitation seasonality (BIO15), but not on PC1 axis (mean divergence = 0.23, null hypothesis = 0.15–0.55; paired samples Student's *t* = 2.437, *df* = 632.84, *p* = .01) including mean diurnal temperature range (BIO2) and precipitation of warmest quarter (BIO18).

**Figure 4:**
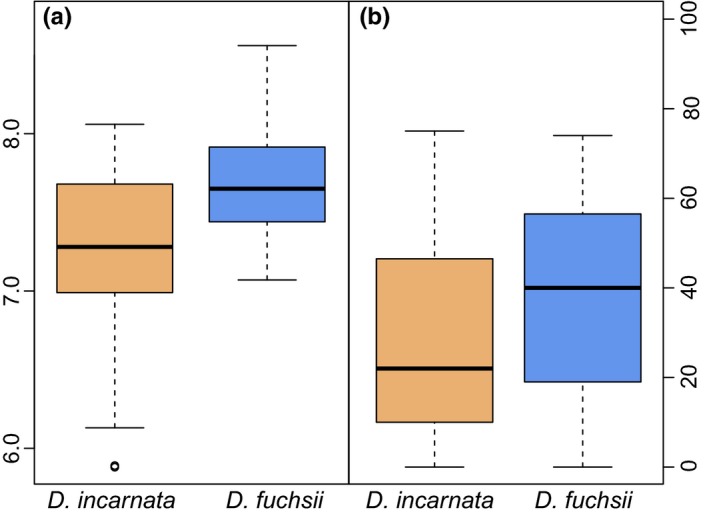
Ecological divergence between *D. fuchsii* and *D. incarnata*. (a) Preference for soil pH measured at 14 populations in Britain, Scandinavia, the Alps and Pyrenees. (b) Preference for tree cover of the two species. The difference in distribution in both cases is significant at *p *<* *.001 [Colour figure can be viewed at wileyonlinelibrary.com]

The tree cover data of 30‐m resolution are considered here a microhabitat descriptor. Significant differences in tree cover were apparent between the localities occupied by the two species (Student's *t* = 6.776, *df* = 641.11, *p* < .001; Figure 4). Another microhabitat descriptor soil pH directly measured in the immediate vicinity of plants indicated a significantly divergent preference for soil acidity (Student's *t* = −3.535, *df* = 62, *p* < .001, Figure 4).

## 4 Discussion

Orchids make up the largest angiosperm family, but the molecular variation shaping this important biodiversity is still little understood (Givnish et al., 2015). Here, we have identified thousands of transcripts with distinct alleles, expression patterns and/or post‐transcriptional regulation between two food‐deceptive, terrestrial orchid species that are currently parapatric across Europe. We show that despite their generally shared distribution area, the two *Dactylorhiza* species inhabit distinct niches, as the microenvironmental conditions they prefer differ significantly with regard to soil acidity and tree cover (Figure 4), but also with respect to temperature evenness over the year and annual precipitation. If maintained over generations, such deviating ecological preferences likely triggered distinct selection that in combination to specific demographic histories have moulded different genomic landscapes between the two species (Slotte, Foxe, Hazzouri, & Wright, 2010).

The current prevalence of *D. incarnata* within small, localized populations (i.e., subhabitat specialization) over a highly fragmented distribution resulting in a Wahlund effect (Hedrén & Nordström, 2009) is corroborated here with considerable levels of inbreeding of this taxon. In turn, *D. fuchsii*, which currently grows in larger, diffuse populations (Kull & Hutchings, 2006), exhibits higher levels of heterozygosity and, in general, higher genetic diversity (Table 2). We estimate the lineages leading to *D. fuchsii* and *D. incarnata* to have diverged in the second half of Miocene, most probably within the first million years after *Dactylorhiza* split from its sister clade (Givnish et al., 2015). An early split of the clades of the two species has previously been demonstrated in phylogenetic (Bateman et al., 2003) and population genetic studies (Balao et al., 2016; Hedrén, 1996) that described significant genetic divergence between them. The two *Dactylorhiza* species studied here can therefore be considered to be representative for the depth of molecular variation in the genus, retaining effects of natural selection throughout their evolutionary histories.

### 4.1 Protein sequence evolution, positive selection and biotic pressure

Nonsynonymous mutations may trigger novel functions and new phenotypes and are hence expected to be under strong selective pressure, either purifying or positive. Along the deep histories of the two *Dactylorhiza* species, we observe significant effects of purifying selection, targeting close to 70% of genes. However, the estimated overall ratio of nonsynonymous to synonymous substitutions of 1:5 appears in line to those recorded for example between *Arabidopsis thaliana* and *A. lyrata* (diverged 5–10 Ma, Yang & Gaut, 2011) or *Gossypium arboreum* and *G. raimondii* (diverged 7–11 Ma, Senchina et al., 2003).

Our results further bring evidence that positive selection elevated the interspecific *K*_a_/*K*_s_ ratios across at least 1.2% of orthologous genes. This estimate is, however, limited to a cohort of protein‐coding sequences that were expressed in both *Dactylorhiza* species at the time of tissue sampling in the common garden and also had an ortholog in the *O. italica* reference. Hence, our inferences may be mainly representative for constitutively expressed genes/exons, rather than those transcribed only as direct response to stimuli or stressors. In addition, given the long history of the two species and therefore the substantial amount of synonymous substitutions that could have accumulated, our test for positive selection requires a fairly large number of codons per gene to be affected by positive selection in order for *K*_a_/*K*_s_ to significantly exceed one. Therefore, the stringent approach we used likely uncovers rather extreme cases where positive selection is operating (Roth & Liberles, 2006).

Consistent with a dynamic evolution of protein sequences shaped by exposure to habitat‐specific pathogens and herbivores (Brown & Tellier, 2011), the most frequent and overrepresented functions among the selected genes along the phylogenetic branches of the two *Dactylorhiza* species are linked to biotic responses. The type and strength of biotic stress imposed on plants depends heavily on both geography and environmental context, but also on host genotype and ability of plants to resist particular stressors (Tiffin & Moeller, 2006). For example, given the open versus more forested habitats specific for *D. incarnata* and *D. fuchsii*, respectively, each of these species had most probably to adapt to distinct herbivores. Plant responses to herbivores and pathogens (i.e., host–pathogens interactions, leading to pathogen recognition and host defence systems; Chisholm, Coaker, Day, & Staskawicz, 2006) are generally context dependent, moulded by the surrounding plant communities, local nutrient conditions and the plant metapopulation dynamics and structure, likely distinct in the long‐term between the two species investigated here (Hedrén & Nordström, 2009; Pillon et al., 2007). Aiming to minimize frequency and impact of attack, the diverse defences that plants specifically evolved appear to include both physical and chemical adaptations. The specific genes that have been found to be under lineage‐specific positive selection with our approach (see Section “2”) and their GO term enriched in the respective test are discussed below.

On the *D. fuchsii* branch (Figure 2a, Fig. S6a), evidence for adaptive evolution is found for a homolog of *DEFENSIN J1‐2*, known to inhibit the growth of pathogenic fungi by modifying their morphology or physiology, disrupting or killing exogenous cells (Thomma, Cammue, & Thevissen, 2002). Another candidate gene for adaptive evolution on the *D. fuchsii* branch is a homolog of *HAI1 PHOSPHATASE 2C 78*, which is involved in defence response mechanisms by deposition of callose, a polysaccharide produced as a response to wounding, but also in response to drought and salt stress (Zhang et al., 2013). *HAI1* is further involved in the pathway of the endoplasmic reticulum response to an accumulation of unfolded or misfolded proteins on stress (Wahyu Indra Duwi, Lee, & Lee, 2013). A *D. fuchsii* homolog of *TETRAKITIDE* α*‐PYRONE REDUCTASE 2* also shows a significantly elevated *K*_a_/*K*_s_ ratio. This is part of the *DIHYDROKAEMPFEROL 4‐REDUCTASE* activity with a role in flavonoid biosynthesis, again potentially linked to plant defence. Finally, a homolog of *3‐KETOACYL‐SYNTHASE 10*, with a role in developing cuticular wax, is also evidenced as a target of positive selection on the *D. fuchsii* branch, potentially in response to viral pressure (Lolle et al., 1997).

On the other hand, along the history of *D. incarnata* (Figure 2b, Fig. S6b), positive selection acted on a *POLYPHENOL OXIDASE* homolog with a putative role in the activity of *CATECHOL OXIDASE* as a response to plant tissue damage, producing ortho‐benzoquinones that further polymerize to form melanins, protecting plant wounds against further microbial attack (Queiroz, Mendes Lopes, Fialho, & Valente‐Mesquita, 2008). Adaptive evolution also appears to have affected a *PRIMARY AMINE OXIDASE* homolog potentially involved in cell wall maturation and lignification during development, as well as wound healing and cell wall reinforcement during pathogen invasion (Cona, Rea, Angelini, Federico, & Tavladoraki, 2006). The *S‐ADENOSYLMETHIONINE SYNTHETASE* orthologs appear to have undergone specific adaptive evolution, independently during both *D. fuchsii* and *D. incarnata* histories. S‐adenosylmethionine acts as a universal methyl group donor in transmethylation reactions, but also serves as a precursor to the phytohormone ethylene, implicated in the control of numerous developmental processes, and regulating resistance to bacterial and fungal pathogens, nematodes and lepidopteran herbivores (Wang, Li, & Ecker, 2002).

Further adaptive evolution specific to the *D. incarnata* history has affected elements of phenylpropanoid biosynthesis, namely a *LACCASE* homolog with a putative role in formation of lignin and a *PHENYLALANINE AMMONIA‐LYASE (PAL)* homolog potentially involved in alkaloid biosynthesis. Apart their role in defence mechanisms against herbivores and pathogens, phenylpropanoids have been shown, for example in the orchid *Phalaenopsis*, to enhance protection to UV light (Ali, Khatun, Hahn, & Paek, 2006), potentially relevant for *D. incarnata* given its preference for more open habitats compared to *D. fuchsii*. In this context, we also detected adaptive evolution along the *D. incarnata* branch of a homolog of *TRANSMEMBRANE 9 SUPERFAMILY MEMBER 4* with a putative role in base excision repair, a cellular DNA repair mechanism.

Elevated evolution of further protein‐coding sequences potentially related to abiotic‐imposed selection in *D. incarnata* includes a *SUGAR CARRIER C* homolog, potentially involved in hexose transmembrane transport likely linked to hypoxia (Gharbi, Ricard, Smiti, Bizid, & Brouquisse, 2009) that the roots of *D. incarnata* may suffer, given the high soil moisture to which this species adapted (Paun et al., 2011). Preferences for distinct soil chemistry (i.e., organic vs. mineral soil typically preferred by *D. incarnata* and *D. fuchsii*, respectively) and soil pH may be linked to elevated rates of evolution of an *INORGANIC PHOSPHATE TRANSPORTER 1‐8* homolog in *D. incarnata* and of *POTASSIUM TRANSPORTER 9* in *D. fuchsii*. Finally, a *MOLYBDATE TRANSPORTER 2* homolog underwent adaptive evolution along the *D. fuchsii* history. Molybdate is an essential plant micronutrient that regulates, for example, nitrate reduction to nitrite and plays a role in redox reactions, and its availability and efficiency of its uptake from soil are highly dependent on soil pH (Tomatsu et al., 2007). Altogether, these candidates open up the ground for further research to identify the specific functional impact of codon evolution, its physiological implications and potential transferability of defence gene variants to cultivated orchids.

### 4.2 Divergent regulation of expression and abiotic preference

We further explored differentiation in gene expression rates between the two *Dactylorhiza* species in a common garden setting. Our results estimate around a third of the identified orthologous genes show significant DE. This extensive expression divergence could be the result of both adaptation and drift and appears mid‐range compared to other pairs of plant species that diverged within a similar time frame (*Brassica rapa* and *B. oleracea* diverged ca. 13 Ma and show ca. 22% DE, Jiang et al., 2013; e.g., *Gossypium* species diverged 5–10 Ma and show up to 53% DE, Rapp, Udall, & Wendel, 2009).

The uncovered DE between the two orchid species affected genes involved in various biosynthetic and metabolic processes—with several GO term enrichments that may be consistent with adaptation (i.e., long‐term constitutive expression divergence) and/or acclimation to multiple abiotic (and biotic) stresses, some of them likely specific for the conditions of the common garden. We further exemplify gene ontology terms of a potential ecological relevance, which have been found to be enriched within the group of DE genes.

The shaded common garden mimicked more closely the light preferences of *D. fuchsii*. Light is one of the most important environmental factors for plants, providing an energy source, but also governing their developmental programme (Neff, Fankhauser, & Chory, 2000). Our tree cover analysis shows a significant difference in preference for the openness of the habitats for *D. incarnata* and *D. fuchsii* (Figure 4b). The need to enhance efficiency of light absorption corroborates an observed overexpression in *D. incarnata* of 14 chlorophyll binding transcripts, part of the light harvesting pathway in photosystem I, which is the most significantly enriched biological process (*p* = 6.2 × 10^−8^) for upregulation in this species (Figure 3b). This further correlates to a significant increase in mi/tasiRNAs targeting of this pathway in *D. incarnata* (i.e., affecting five chlorophyll binding transcripts; enrichment *p* = 3.1 × 10^−4^). This may indicate a dynamic regulation of acclimation to shaded conditions in *D. incarnata*. Further gene expression differences potentially increasing photosynthetic efficiency, this time in the way light is used to reduce molecules in light reaction, are visible in 63 transcripts overexpressed in *D. fuchsii* (or with decreased expression in *D. incarnata*, corresponding also to increased targeting by mi/tasiRNAs of 15 transcripts in this pathway, enriched at *p* = 1.1 × 10^−4^). Broadly, transcripts with a putative role in photosynthesis appear to have higher expression (Figure 3a; enriched at *p* = 1.6 × 10^−10^) and to be less targeted by mi/tasiRNAs (Fig. S9b; enriched at *p* = 6.1 × 10^−7^) in *D. fuchsii* in comparison with *D. incarnata*—a pattern that may mirror the long‐term preference of the former for more shaded environments.

In turn, the location of the common garden has likely featured rather alien temperature conditions for *D. fuchsii* (i.e., higher temperature in summer, limited snow cover in winter), given that no population of this species grows within at least 50 km radius around the city, whereas some *D. incarnata* stands are present even within the city boundary (e.g., compare distributions on www.gbif.at). Indeed, for *D. fuchsii* we identified a significant overexpression of 55 annotated transcripts putatively involved in a response to temperature (enriched at *p* = .0011). This abiotic stress can trigger misfolding of newly synthesized proteins and denaturation of existing proteins (Barnabas, Jäger, & Feher, 2008). Genes known to be upregulated as response to high temperature stress (e.g., Altenbach, 2012) and having a homolog found to be overexpressed in *D. fuchsii* include, for example, heat‐shock proteins (i.e., *HSP70 kDa17* and *DNAJ HSP40* homologs), a *GLYCERALDEHYDE‐3‐PHOSPHATE DEHYDROGENASE* (*GADPH A*), a *GALACTINOL SYNTHASE* and a *TRIOSEPHOSPHATE ISOMERASE* homolog involved in glycolysis. The uncovered ecological divergence in bioclimatic variables such as annual mean temperature (BIO1) is congruent with a significant divergence in expression of genes related to a temperature response.

### 4.3 Genome stability and small RNA regulation

Gene expression is generally governed by a complex combination of regulators, including among others transcription factors, splicing factors and miRNAs (Warnefors & Eyre‐Walker, 2011). The comparative analysis of sRNA profiles confirms that the extensive differences in gene expression between the *Dactylorhiza* species are complemented by divergent post‐transcriptional regulations. miRNAs function as negative regulators of gene expression via translational repression or mRNA cleavage (Borges & Martienssen, 2015) and are known to mediate response to genomic or environmental stresses (Ding, Tao, & Zhu, 2013; Ivashuta et al., 2011). From the transcripts that were targeted by miRNAs and/or tasiRNAs, 27.2% were found to be differentially regulated between the two orchid species, with deviating post‐transcriptional regulation of metabolism (e.g., nucleic acids, aromatic compounds, tetrapyrrole and alkaloid metabolism targeted in *D. fuchsii*, and DNA and tetrapyrrole metabolism targeted in *D. incarnata*) and binding (Fig. S9). As discussed above, we observed an increased targeting by mi/tasiRNA of photosynthetic pathways in *D. incarnata*, potentially as an acclimation response to the shaded conditions in the common garden. Likely in connection to its photosynthetic activity, the starch transitory breakdown including maltose metabolism is also overregulated in *D. incarnata*. Starch represents an overflow of photosynthetically fixed carbon that is exported from chloroplasts at night, predominantly as maltose (Lu & Sharkey, 2006). Inhibition of maltose metabolism slows down starch degradation, which has been reported to be a response to changes in photoperiod length, circadian clock and temperature (Lu & Sharkey, 2006).

Importantly, our results point to distinct strategies and efficiency in the maintenance of genome stability between the two orchid species. In the RNAseq data, RdDp activity (enriched at *p* = .0016; with 60 transcripts overexpressed, representing in particular Ty3‐gypsy subclass), mainly associated with retrotransposons and retroviruses, together with the process of DNA integration (193 transcripts, enriched at *p* = 9.1 × 10^−6^) appear to be overexpressed in *D. incarnata* (Figure 3, Fig. S8). Moreover, the RdDp activity shows increased control by mi/tasiRNAs in *D. fuchsii* (20 transcripts, enriched at *p* = .003; Fig. S9c). These suggest a significant increase in activity of class I TEs in *D. incarnata* compared to *D. fuchsii*, which over time may have triggered their difference in genome size. In this context, it is notable that the two species exhibit different effective population sizes (Balao et al., 2016), reflected in higher inbreeding in *D. incarnata* that may correlate also with less efficient purging of active insertions of TEs.

On the other hand, we also detect divergent regulation of 31.7% of the transcripts that are targeted by siRNAs in the two *Dactylorhiza* species. GO enrichment analyses of overregulated siRNAs for each species revealed that the most meaningful biological processes appear related to DNA integration, DNA metabolism and other processes related to nucleic acid binding (Fig. S10). These biological processes are congruent with the presumed defence function of siRNAs in plants against the proliferation of endogenous TEs or invading viruses through transcriptional or post‐transcriptional gene silencing (Carrington & Ambros, 2003). However, siRNAs could also indirectly affect gene expression of adjacent genes in the proximity of TEs (Hollister et al., 2011). Thus, changes in expression in sRNAs could ultimately be adaptive and evolve quickly under natural selection (Chapman & Carrington, 2007).

### 4.4 Implications

Altogether, the distinct responses to selection accumulated during the history of the two parapatric *Dactylorhiza* species investigated here appear governed by their divergent microenvironmental context, in which biotic and abiotic pressures act synergistically to shape transcriptome structure, expression and regulation. Apart from adaptation to divergent abiotic conditions, distinct habitat affiliation of nascent species requires divergent adaptation to abiotic conditions, if they exceed possibilities granted by phenotypic plasticity. However, distinct microhabitat preferences readily require divergent defence strategies in order to detect and respond to specific pathogens and herbivores (e.g., Turcotte, Davies, Thomsen, & Johnson, 2014). The divergent mechanisms of biotic responses apparent between the two *Dactylorhiza* species both at the level of coding sequence evolution, but also in the rates of expression across the systemic acquired resistance (upregulated in *D. fuchsii*, enriched at *p *<* *.005) and the induced systemic resistance (overexpressed in *D. incarnata*, enriched at *p *<* *.01), are remarkable given their obligate orchid–mycorrhiza interactions (Tupac Otero & Flanagan, 2006). Modulation of plant defence responses could have detrimental secondary effects on their symbiotic relationship. On the other hand, the plant mechanism of mycorrhiza recognition may constitutively activate systemic plant immune responses (Pozo, Verhage, García‐Andrade, García, & Azcón‐Aguilar, 2009). Such activation can lead to a primed state of the plant that allows for a more efficient defence mechanisms in response to attack by potential enemies. Although *Dactylorhiza* species associate with a wide range of common and generalist mycorrhizal fungi, these two species show an incipient differentiation in mycorrhizal spectra (Jacquemyn, Deja, De hert, Cachapa Bailarote, & Lievens, 2012).

The detailed transcriptome knowledge for *Dactylorhiza* gathered in this study provides critical resources to understand evolution after hybridization and whole‐genome duplication, phenomena especially frequent in this genus. In particular, the hybridization of the two species analysed here iteratively formed a myriad of morphologically and ecologically distinct sibling allopolyploids (Pillon et al., 2007). We demonstrate here an extensive molecular divergence between the two diploids, which suggests an extensive genomic and transcriptomic shock in their hybrids (McClintock, 1984) and offers potential insights into the difficulties of coexistence for these two genomes at the homoploid level (Paun, Forest, Fay, & Chase, 2009). In turn, whole‐genome duplication was proposed to alleviate the transcriptomic shock of hybridization between divergent progenitors (Hegarty et al., 2006). The present study hence contributes useful resources and background information to further explore allelic interactions between parental genomes when combined in a single duplicated nucleus. This notably leads to altered reprogramming of gene expression, including dominance and transgressive patterns that may promote metabolic and developmental changes, stress tolerance and increased fitness in allopolyploids (Chen, 2013).

## Data Accessibility

The raw RNAseq data have been deposited into the Short Read Archive (SRA) of the NCBI under Accession nos. SRR3330392–SRR3330400 and SRR3330403–SRR3330404. The raw sRNA data files have been deposited into the NCBI SRA under Accession nos. SRR3331132–SRR3331133, SRR3331136, SRR3331143, SRR3331149–SRR3331151, SRR3331195 and SRR5365231. The Transcriptome Shotgun Assembly project has been deposited at DDBJ/EMBL/GenBank under the Accession nos. GFHX00000000 and GFJV00000000.

## Author Contributions

OP designed the research. F.B., M.H. and O.P. collected the sample material. M.T.L. and J.B. carried out the wet laboratory work with advice from O.P. and L.S. B.H.H. assembled the reference genome. F.B. and T.W. carried out the D.E. analyses, with input from F.A. O.P. performed the cSNP analyses, with input from C.K. E.T. performed the sRNA analyses. F.B. carried out the ecological analyses. M.W.C. and M.H. contributed to data interpretation. O.P., F.B. and E.T. drafted the manuscript. All authors revised and approved the final manuscript.

## References

[mec14123-bib-0001] Aagaard, S. M. , Sastad, S. M. , Greilhuber, J. , & Moen, A. (2005). A secondary hybrid zone between diploid *Dactylorhiza incarnata* ssp. *cruenta* and allotetraploid *D. lapponica* (Orchidaceae). Heredity, 94, 488–496. 15785782 10.1038/sj.hdy.6800643

[mec14123-bib-0002] Ackerman, J. D. , Trejo‐Torres, J. C. , & Crespo‐Chuy, Y. (2007). Orchids of the West Indies: Predictability of diversity and endemism. Journal of Biogeography, 34, 779–786.

[mec14123-bib-0003] Ali, M. B. , Khatun, S. , Hahn, E.‐J. , & Paek, K.‐Y. (2006). Enhancement of phenylpropanoid enzymes and lignin in Phalaenopsis orchid and their influence on plant acclimatisation at different levels of photosynthetic photon flux. Plant Growth Regulation, 49, 137–146.

[mec14123-bib-0004] Altenbach, S. B. (2012). New insights into the effects of high temperature, drought and post‐anthesis fertilizer on wheat grain development. Journal of Cereal Science, 56, 39–50.

[mec14123-bib-0005] Anders, S. , & Huber, W. (2010). Differential expression analysis for sequence count data. Genome Biology, 11, R106. 20979621 10.1186/gb-2010-11-10-r106 PMC3218662

[mec14123-bib-0006] Anderson, J. T. , Wagner, M. R. , Rushworth, C. A. , Prasad, K. V. S. K. , & Mitchell‐Olds, T. (2014). The evolution of quantitative traits in complex environments. Heredity, 112, 4–12. 23612691 10.1038/hdy.2013.33 PMC3860162

[mec14123-bib-0007] Balao, F. , Tannhäuser, M. , Lorenzo, M. T. , Hedren, M. , & Paun, O. (2016). Genetic differentiation and admixture between sibling allopolyploids in the *Dactylorhiza majalis* complex. Heredity, 116, 351–361. 26604189 10.1038/hdy.2015.98 PMC4787024

[mec14123-bib-0008] Barnabas, B. , Jäger, K. , & Feher, A. (2008). The effect of drought and heat stress on reproductive processes in cereals. Plant, Cell & Environment, 31, 11–38. 10.1111/j.1365-3040.2007.01727.x 17971069

[mec14123-bib-0009] Bateman, R. M. , Hollingsworth, P. M. , Preston, J. , Luo, Y. B. , Pridgeon, A. M. , & Chase, M. W. (2003). Molecular phylogenetics and evolution of Orchidinae and selected Habenariinae (Orchidaceae). Botanical Journal of the Linnean Society, 142, 1–40.

[mec14123-bib-0010] Benzing, D. H. (1986). The genesis of orchid diversity: Emphasis on floral biology leads to misconceptions. Linleyana, 1, 73–89.

[mec14123-bib-0011] Borges, F. , & Martienssen, R. A. (2015). The expanding world of small RNAs in plants. Nature Reviews Molecular Cell Biology, 16, 727–741. 26530390 10.1038/nrm4085 PMC4948178

[mec14123-bib-0012] Broennimann, O. , Fitzpatrick, M. C. , Pearman, P. B. , Petitpierre, B. , Pellissier, L. , & Yoccoz, N. G. , … Guisan, A . (2012). Measuring ecological niche overlap from occurrence and spatial environmental data. Global Ecology and Biogeography, 21, 481–497.

[mec14123-bib-0013] Brown, J. K. M. , & Tellier, A. (2011). Plant‐parasite coevolution: Bridging the gap between genetics and ecology. Annual Review of Phytopathology, 49, 345–367. 10.1146/annurev-phyto-072910-095301 21513455

[mec14123-bib-0014] Cai, J. , Liu, X. , Vanneste, K. , Proost, S. , Tsai, W. ‐C. , & Liu, K. ‐W. , … Liu, Z. ‐J . (2015). The genome sequence of the orchid *Phalaenopsis equestris* . Nature Genetics, 47, 65–72. 25420146 10.1038/ng.3149

[mec14123-bib-0015] Carrington, J. C. , & Ambros, V. (2003). Role of MicroRNAs in plant and animal development. Science, 301, 336–338. 12869753 10.1126/science.1085242

[mec14123-bib-0016] Chapman, E. J. , & Carrington, J. C. (2007). Specialization and evolution of endogenous small RNA pathways. Nature Reviews Genetics, 8, 884–896. 10.1038/nrg2179 17943195

[mec14123-bib-0017] Chase, M. W. , Cameron, K. M. , Freudenstein, J. V. , Pridgeon, A. M. , Salazar, G. , vanden Berg, C. , & Schuiteman, A . (2015). An updated classification of Orchidaceae. Botanical Journal of the Linnean Society, 177, 151–174.

[mec14123-bib-0018] Chen, Z. J. (2013). Genomic and epigenetic insights into the molecular bases of heterosis. Nature Reviews Genetics, 14, 471–482. 10.1038/nrg3503 23752794

[mec14123-bib-0019] Chisholm, S. T. , Coaker, G. , Day, B. , & Staskawicz, B. J. (2006). Host‐microbe interactions: Shaping the evolution of the plant immune response. Cell, 124, 803–814. 16497589 10.1016/j.cell.2006.02.008

[mec14123-bib-0020] Cona, A. , Rea, G. , Angelini, R. , Federico, R. , & Tavladoraki, P. (2006). Functions of amine oxidases in plant development and defence. Trends in Plant Science, 11, 80–88. 16406305 10.1016/j.tplants.2005.12.009

[mec14123-bib-0021] Conesa, A. , & Götz, S. (2008). Blast2GO: A comprehensive suite for functional analysis in plant genomics. International Journal of Plant Genomics, 2008, 12. 10.1155/2008/619832 PMC2375974 18483572

[mec14123-bib-0022] Cozzolino, S. , & Widmer, A. (2005). Orchid diversity: An evolutionary consequence of deception? Trends in Ecology & Evolution, 20, 487–494. 16701425 10.1016/j.tree.2005.06.004

[mec14123-bib-0023] Danecek, P. , Auton, A. , Abecasis, G. , Albers, C. A. , Banks, E. , & DePristo, M. A. , … Durbin, R . (2011). The variant call format and VCFtools. Bioinformatics, 27, 2156–2158. 21653522 10.1093/bioinformatics/btr330 PMC3137218

[mec14123-bib-0024] Darwin, C. (1877). The various contrivances by which orchids are fertilized by insects. London: John Murray.

[mec14123-bib-0025] Davidson, N. M. , & Oshlack, A. (2014). Corset: Enabling differential gene expression analysis for de novo assembled transcriptomes. Genome Biology, 15, 410. 25063469 10.1186/s13059-014-0410-6 PMC4165373

[mec14123-bib-0026] De Paolo, S. , Salvemini, M. , Gaudio, L. , & Aceto, S. (2014). *De novo* transcriptome assembly from inflorescence of *Orchis italica*: Analysis of coding and non‐coding transcripts. PLoS One, 9, e102155. 25025767 10.1371/journal.pone.0102155 PMC4099010

[mec14123-bib-0027] De Wit, P. , Pespeni, M. H. , & Palumbi, S. R. (2015). SNP genotyping and population genomics from expressed sequences—Current advances and future possibilities. Molecular Ecology, 24, 2310–2323. 25808983 10.1111/mec.13165

[mec14123-bib-0028] DePristo, M. A. , Banks, E. , Poplin, R. E. , Garimella, K. V. , Maguire, J. R. , & Hartl, C. , … Daly, M. J. (2011). A framework for variation discovery and genotyping using next‐generation DNA sequencing data. Nature Genetics, 43, 491–498. 21478889 10.1038/ng.806 PMC3083463

[mec14123-bib-0029] Devos, N. , Raspe, O. , Oh, S. H. , Tyteca, D. , & Jacquemart, A. L. (2006). The evolution of *Dactylorhiza* (Orchidaceae) allotetraploid complex: Insights from nrDNA sequences and cpDNA PCR‐RFLP data. Molecular Phylogenetics and Evolution, 38, 767–778. 16439164 10.1016/j.ympev.2005.11.013

[mec14123-bib-0030] Ding, Y. , Tao, Y. , & Zhu, C. (2013). Emerging roles of microRNAs in the mediation of drought stress response in plants. Journal of Experimental Botany, 64, 3077–3086. 23814278 10.1093/jxb/ert164

[mec14123-bib-0031] Dobin, A. , Davis, C. A. , Schlesinger, F. , Drenkow, J. , Zaleski, C. , & Jha, S. , … Gingeras, T. R. (2013). STAR: Ultrafast universal RNA‐seq aligner. Bioinformatics, 29, 15–21. 23104886 10.1093/bioinformatics/bts635 PMC3530905

[mec14123-bib-0032] Ehrenreich, I. M. , & Purugganan, M. D. (2008). Sequence variation of MicroRNAs and their binding sites in *Arabidopsis* . Plant Physiology, 146, 1974–1982. 18305205 10.1104/pp.108.116582 PMC2287364

[mec14123-bib-0033] Engström, P. G. , Steijger, T. , Sipos, B. , Grant, G. R. , Kahles, A. , & Rätsch, G. , … Bertone, P. (2013). Systematic evaluation of spliced alignment programs for RNA‐seq data. Nature Methods, 10, 1185–1191. 24185836 10.1038/nmeth.2722 PMC4018468

[mec14123-bib-0034] Gharbi, I. , Ricard, B. , Smiti, S. , Bizid, E. , & Brouquisse, R. (2009). Increased hexose transport in the roots of tomato plants submitted to prolonged hypoxia. Planta, 230, 441–448. 19437034 10.1007/s00425-009-0941-3

[mec14123-bib-0035] Givnish, T. J. , Spalink, D. , Ames, M. , Lyon, S. P. , Hunter, S. J. , & Zuluaga, A. , … Cameron, K. M. (2015). Orchid phylogenomics and multiple drivers of their extraordinary diversification. Proceedings of the Royal Society of London B: Biological Sciences, 282, 20151553. 10.1098/rspb.2015.1553 PMC4571710 26311671

[mec14123-bib-0036] Gravendeel, B. , Smithson, A. , Slik, F. J. W. , & Schuiteman, A. (2004). Epiphytism and pollinator specialization: Drivers for orchid diversity?. Philosophical Transactions of the Royal Society of London Series B: Biological Sciences, 359, 1523–1535. 15519970 10.1098/rstb.2004.1529 PMC1693444

[mec14123-bib-0037] Ha, M. , Pang, M. , Agarwal, V. , & Chen, Z. J. (2008). Interspecies regulation of microRNAs and their targets. Biochimica et Biophysica Acta, 1779, 735–742. 18407843 10.1016/j.bbagrm.2008.03.004 PMC2586835

[mec14123-bib-0038] Haas, B. J. , Papanicolaou, A. , Yassour, M. , Grabherr, M. , Blood, P. D. , & Bowden, J. , … Regev, A . (2013). De novo transcript sequence reconstruction from RNA‐seq using the Trinity platform for reference generation and analysis. Nature Protocols, 8, 1494–1512. 23845962 10.1038/nprot.2013.084 PMC3875132

[mec14123-bib-0039] Hardcastle, T. J. , & Kelly, K. A. (2010). baySeq: Empirical Bayesian methods for identifying differential expression in sequence count data. BMC Bioinformatics, 11, 422. 20698981 10.1186/1471-2105-11-422 PMC2928208

[mec14123-bib-0040] Hedrén, M. (1996). Genetic differentiation, polyploidization and hybridization in northern European *Dactylorhiza* (Orchidaceae): Evidence from allozymes markers. Plant Systematics and Evolution, 201, 31–55.

[mec14123-bib-0041] Hedrén, M. , Fay, M. F. , & Chase, M. W. (2001). Amplified fragment length polymorphisms (AFLP) reveal details of polyploid evolution in *Dactylorhiza* (Orchidaceae). American Journal of Botany, 88, 1868–1880. 21669620

[mec14123-bib-0042] Hedrén, M. , & Nordström, S. (2009). Polymorphic populations of *Dactylorhiza incarnata* s.l. (Orchidaceae) on the Baltic island of Gotland: Morphology, habitat preference and genetic differentiation. Annals of Botany, 104, 527–542. 19458026 10.1093/aob/mcp102 PMC2720652

[mec14123-bib-0043] Hegarty, M. J. , Barker, G. L. , Wilson, I. D. , Abbott, R. J. , Edwards, K. J. , & Hiscock, S. J. (2006). Transcriptome shock after interspecific hybridization in *Senecio* is ameliorated by genome duplication. Current Biology, 16, 1652–1659. 16920628 10.1016/j.cub.2006.06.071

[mec14123-bib-0044] Hollister, J. D. , Smith, L. M. , Guo, Y.‐L. , Ott, F. , Weigel, D. , & Gaut, B. S. (2011). Transposable elements and small RNAs contribute to gene expression divergence between *Arabidopsis thaliana* and *Arabidopsis lyrata* . Proceedings of the National Academy of Sciences of the United States of America, 108, 2322–2327. 21252301 10.1073/pnas.1018222108 PMC3038775

[mec14123-bib-0045] Huber, W. , Carey, V. J. , Gentleman, R. , Anders, S. , Carlson, M. , Carvalho, B. S. , … Morgan, M. (2015). Orchestrating high‐throughput genomic analysis with bioconductor. Nature Methods, 12, 115–121. 25633503 10.1038/nmeth.3252 PMC4509590

[mec14123-bib-0046] Inda, L. A. , Pimentel, M. , & Chase, M. W. (2012). Phylogenetics of tribe Orchideae (Orchidaceae: Orchidoideae) based on combined DNA matrices: Inferences regarding timing of diversification and evolution of pollination syndromes. Annals of Botany, 110, 71–90. 22539542 10.1093/aob/mcs083 PMC3380586

[mec14123-bib-0047] Ivashuta, S. , Banks, I. R. , Wiggins, B. E. , Zhang, Y. , Ziegler, T. E. , Roberts, J. K. , & Heck, G. R. (2011). Regulation of gene expression in plants through miRNA inactivation. PLoS One, 6, e21330. 21731706 10.1371/journal.pone.0021330 PMC3121747

[mec14123-bib-0048] Jacquemyn, H. , Deja, A. , De hert, K. , Cachapa Bailarote, B. , & Lievens, B. (2012). Variation in mycorrhizal associations with tulasnelloid fungi among populations of five *Dactylorhiza* species. PLoS One, 7, e42212. 22870305 10.1371/journal.pone.0042212 PMC3411701

[mec14123-bib-0049] Jiang, J. , Shao, Y. , Du, K. , Ran, L. , Fang, X. , & Wang, Y. (2013). Use of digital gene expression to discriminate gene expression differences in early generations of resynthesized *Brassica napus* and its diploid progenitors. BMC Genomics, 14, 72. 23369045 10.1186/1471-2164-14-72 PMC3608150

[mec14123-bib-0050] Kitano, J. , Yoshida, K. , & Suzuki, Y. (2013). RNA sequencing reveals small RNAs differentially expressed between incipient Japanese threespine sticklebacks. BMC Genomics, 14, 1–13. 23547919 10.1186/1471-2164-14-214 PMC3637797

[mec14123-bib-0051] Kull, T. , & Hutchings, M. J. (2006). A comparative analysis of decline in the distribution ranges of orchid species in Estonia and the United Kingdom. Biological Conservation, 129, 31–39.

[mec14123-bib-0052] Lischer, H. E. L. , & Excoffier, L. (2012). PGDSpider: An automated data conversion tool for connecting population genetics and genomics programs. Bioinformatics, 28, 298–299. 22110245 10.1093/bioinformatics/btr642

[mec14123-bib-0053] Lolle, S. J. , Berlyn, G. P. , Engstrom, E. M. , Krolikowski, K. A. , Reiter, W. D. , & Pruitt, R. E. (1997). Developmental regulation of cell interactions in the Arabidopsis fiddlehead‐1 mutant: A role for the epidermal cell wall and cuticle. Developmental Biology, 189, 311–321. 9299123 10.1006/dbio.1997.8671

[mec14123-bib-0054] Lu, Y. A. N. , & Sharkey, T. D. (2006). The importance of maltose in transitory starch breakdown. Plant, Cell & Environment, 29, 353–366. 10.1111/j.1365-3040.2005.01480.x 17080591

[mec14123-bib-0055] McClintock, B. (1984). The significance of responses of the genome to challenge. Science, 226, 792–801. 15739260 10.1126/science.15739260

[mec14123-bib-0056] McCormack, J. E. , Zellmer, A. J. , & Knowles, L. L. (2010). Does niche divergence accompany allopatric divergence in *Aphelocoma jays* as predicted under ecological speciation? Insights from tests with niche models. Evolution, 64, 1231–1244. 19922442 10.1111/j.1558-5646.2009.00900.x

[mec14123-bib-0057] McKenna, A. , Hanna, M. , Banks, E. , Sivachenko, A. , Cibulskis, K. , Kernytsky, A. , … DePristo, M. A. (2010). The Genome Analysis Toolkit: A MapReduce framework for analyzing next‐generation DNA sequencing data. Genome Research, 20, 1297–1303. 20644199 10.1101/gr.107524.110 PMC2928508

[mec14123-bib-0058] McManus, C. J. , Coolon, J. D. , Duff, M. O. , Eipper‐Mains, J. , Graveley, B. R. , & Wittkopp, P. J. (2010). Regulatory divergence in *Drosophila* revealed by mRNA‐seq. Genome Research, 20, 816–825. 20354124 10.1101/gr.102491.109 PMC2877578

[mec14123-bib-0059] Michalak, P. (2008). Epigenetic, transposon and small RNA determinants of hybrid dysfunctions. Heredity, 102, 45–50. 18545265 10.1038/hdy.2008.48

[mec14123-bib-0060] Moccia, M. D. , Widmer, A. , & Cozzolino, S. (2007). The strength of reproductive isolation in two hybridizing food‐deceptive orchid species. Molecular Ecology, 16, 2855–2866. 17614902 10.1111/j.1365-294X.2007.03240.x

[mec14123-bib-0061] Neff, M. M. , Fankhauser, C. , & Chory, J. (2000). Light: An indicator of time and place. Genes & Development, 14, 257–271. 10673498

[mec14123-bib-0062] Nielsen, R. (2005). Molecular signatures of natural selection. Annual Review of Genetics, 39, 197–218. 10.1146/annurev.genet.39.073003.112420 16285858

[mec14123-bib-0063] Paun, O. , Bateman, R. M. , Fay, M. F. , Luna, J. A. , Moat, J. , Hedrén, M. , & Chase, M. W. (2011). Altered gene expression and ecological divergence in sibling allopolyploids of *Dactylorhiza* (Orchidaceae). BMC Evolutionary Biology, 11, 113. 21521507 10.1186/1471-2148-11-113 PMC3112086

[mec14123-bib-0064] Paun, O. , Forest, F. , Fay, M. F. , & Chase, M. W. (2009). Hybrid speciation in angiosperms: Parental divergence drives ploidy. New Phytologist, 182, 507–518. 19220761 10.1111/j.1469-8137.2009.02767.x PMC2988484

[mec14123-bib-0065] Pillon, Y. , Fay, M. F. , Hedrén, M. , Bateman, R. M. , Devey, D. S. , Shipunov, A. B. , … Chase, M. W. (2007). Evolution and temporal diversification of western European polyploid species complexes in *Dactylorhiza* (Orchidaceae). Taxon, 56, 1185–1208.

[mec14123-bib-0066] Pozo, M. , Verhage, A. , García‐Andrade, J. , García, J. , & Azcón‐Aguilar, C. (2009). Priming plant defence against pathogens by arbuscular mycorrhizal fungi In Azcón‐Aguilar C., Barea J., Gianinazzi S., & Gianinazzi‐Pearson V. (Eds.), Mycorrhizas‐functional processes and ecological impact (pp. 123–135). Berlin Heidelberg: Springer.

[mec14123-bib-0067] Queiroz, C. , Mendes Lopes, M. L. , Fialho, E. , & Valente‐Mesquita, V. L. (2008). Polyphenol oxidase: Characteristics and mechanisms of browning control. Food Reviews International, 24, 361–375.

[mec14123-bib-0068] Quinlan, A. R. , & Hall, I. M. (2010). BEDTools: A flexible suite of utilities for comparing genomic features. Bioinformatics, 26, 841–842. 20110278 10.1093/bioinformatics/btq033 PMC2832824

[mec14123-bib-0069] Rapp, R. A. , Udall, J. A. , & Wendel, J. F. (2009). Genomic expression dominance in allopolyploids. BMC Biology, 7, 18. 19409075 10.1186/1741-7007-7-18 PMC2684529

[mec14123-bib-0070] Risso, D. , Ngai, J. , Speed, T. P. , & Dudoit, S. (2014). Normalization of RNA‐seq data using factor analysis of control genes or samples. Nature Biotechnology, 32, 896–902. 10.1038/nbt.2931 PMC4404308 25150836

[mec14123-bib-0071] Robinson, M. D. , McCarthy, D. J. , & Smyth, G. K. (2010). edgeR: A Bioconductor package for differential expression analysis of digital gene expression data. Bioinformatics, 26, 139–140. 19910308 10.1093/bioinformatics/btp616 PMC2796818

[mec14123-bib-0072] Roth, C. , & Liberles, D. A. (2006). A systematic search for positive selection in higher plants (Embryophytes). BMC Plant Biology, 6, 12. 16784532 10.1186/1471-2229-6-12 PMC1540423

[mec14123-bib-0073] Schlicker, A. , Domingues, F. S. , Rahnenführer, J. , & Lengauer, T. (2006). A new measure for functional similarity of gene products based on Gene Ontology. BMC Bioinformatics, 7, 302. 16776819 10.1186/1471-2105-7-302 PMC1559652

[mec14123-bib-0074] Schoener, T. W. (1970). Nonsynchronous spatial overlap of lizards in patchy habitats. Ecology, 51, 408–418.

[mec14123-bib-0075] Senchina, D. S. , Alvarez, I. , Cronn, R. C. , Liu, B. , Rong, J. , Noyes, R. D. , … Wendel, J. F . (2003). Rate variation among nuclear genes and the age of polyploidy in *Gossypium* . Molecular Biology and Evolution, 20, 633–643. 12679546 10.1093/molbev/msg065

[mec14123-bib-0076] Silvera, K. , Santiago, L. S. , Cushman, J. C. , & Winter, K. (2009). Crassulacean acid metabolism and epiphytism linked to adaptive radiations in the Orchidaceae. Plant Physiology, 149, 1838–1847. 19182098 10.1104/pp.108.132555 PMC2663729

[mec14123-bib-0077] Sletvold, N. , Grindeland, J. M. , & Ågren, J. (2010). Pollinator‐mediated selection on floral display, spur length and flowering phenology in the deceptive orchid *Dactylorhiza lapponica* . New Phytologist, 188, 385–392. 20497348 10.1111/j.1469-8137.2010.03296.x

[mec14123-bib-0078] Slotte, T. , Foxe, J. P. , Hazzouri, K. M. , & Wright, S. I. (2010). Genome‐wide evidence for efficient positive and purifying selection in *Capsella grandiflora*, a plant species with a large effective population size. Molecular Biology and Evolution, 27, 1813–1821. 20194429 10.1093/molbev/msq062

[mec14123-bib-0079] Stamatakis, A. (2014). RAxML version 8: A tool for phylogenetic analysis and post‐analysis of large phylogenies. Bioinformatics, 30, 1312–1313. 24451623 10.1093/bioinformatics/btu033 PMC3998144

[mec14123-bib-0080] Supek, F. , Bošnjak, M. , Škunca, N. , & Šmuc, T. (2011). REVIGO summarizes and visualizes long lists of gene ontology terms. PLoS One, 6, e21800. 21789182 10.1371/journal.pone.0021800 PMC3138752

[mec14123-bib-0081] Thomma, B. P. , Cammue, B. P. , & Thevissen, K. (2002). Plant defensins. Planta, 216, 193–202. 12447532 10.1007/s00425-002-0902-6

[mec14123-bib-0082] Tiffin, P. , & Moeller, D. A. (2006). Molecular evolution of plant immune system genes. Trends in Genetics, 22, 662–670. 17011664 10.1016/j.tig.2006.09.011

[mec14123-bib-0083] Tomatsu, H. , Takano, J. , Takahashi, H. , Watanabe‐Takahashi, A. , Shibagaki, N. , & Fujiwara, T. (2007). An *Arabidopsis thaliana* high‐affinity molybdate transporter required for efficient uptake of molybdate from soil. Proceedings of the National Academy of Sciences of the United States of America, 104, 18807–18812. 18003916 10.1073/pnas.0706373104 PMC2141858

[mec14123-bib-0084] Tremblay, R. L. , Ackerman, J. D. , Zimmerman, J. K. , & Calvo, R. N. (2005). Variation in sexual reproduction in orchids and its evolutionary consequences: A spasmodic journey to diversification. Biological Journal of the Linnean Society, 84, 1–54.

[mec14123-bib-0085] Tupac Otero, J. , & Flanagan, N. S. (2006). Orchid diversity—beyond deception. Trends in Ecology & Evolution, 21, 64–65. 16701475 10.1016/j.tree.2005.11.016

[mec14123-bib-0086] Turcotte, M. M. , Davies, T. J. , Thomsen, C. J. M. , & Johnson, M. T. J. (2014). Macroecological and macroevolutionary patterns of leaf herbivory across vascular plants. Proceedings of the Royal Society of London B: Biological Sciences, 281, 20140555. 10.1098/rspb.2014.0555 PMC4071545 24870043

[mec14123-bib-0087] Tutin, T. , Heywood, V. , Moore, D. M. , Valentine, D. H. , Walters, S. M. , & Webb, D. A. (1980). Flora Europaea, volume 5: Alismataceae to Orchidaceaea (Monocotyleones). Cambridge: Cambridge University Press.

[mec14123-bib-0088] Van der Auwera, G. A. , Carneiro, M. O. , Hartl, C. Poplin, R. , Levy‐moonshine, A. , Jordan, T. , … Depristo, M. A. (2013). From FastQ data to high‐confidence variant calls: The genome analysis toolkit best practices pipeline Current Protocols in Bioinformatics 43 11.10.1‐11.10.33. 10.1002/0471250953.bi1110s43 PMC4243306 25431634

[mec14123-bib-0089] Wahyu Indra Duwi, F. , Lee, S. Y. , & Lee, K. O. (2013). The unfolded protein response in plants: A fundamental adaptive cellular response to internal and external stresses. Journal of Proteomics, 93, 356–368. 23624343 10.1016/j.jprot.2013.04.023

[mec14123-bib-0090] Wang, K. L.‐C. , Li, H. , & Ecker, J. R. (2002). Ethylene biosynthesis and signaling networks. The Plant Cell, 14, S131–S151. 12045274 10.1105/tpc.001768 PMC151252

[mec14123-bib-0091] Warnefors, M. , & Eyre‐Walker, A. (2011). The accumulation of gene regulation through time. Genome Biology and Evolution, 3, 667–673. 21398425 10.1093/gbe/evr019 PMC3157833

[mec14123-bib-0092] Wong, W. S. W. , Yang, Z. , Goldman, N. , & Nielsen, R. (2004). Accuracy and power of statistical methods for detecting adaptive evolution in protein coding sequences and for identifying positively selected sites. Genetics, 168, 1041–1051. 15514074 10.1534/genetics.104.031153 PMC1448811

[mec14123-bib-0093] Wray, G. A. (2007). The evolutionary significance of cis‐regulatory mutations. Nature Reviews Genetics, 8, 206–216. 10.1038/nrg2063 17304246

[mec14123-bib-0094] Yang, Z. (2007). PAML 4: Phylogenetic analysis by maximum likelihood. Molecular Biology and Evolution, 24, 1586–1591. 17483113 10.1093/molbev/msm088

[mec14123-bib-0095] Yang, L. , & Gaut, B. S. (2011). Factors that contribute to variation in evolutionary rate among *Arabidopsis* genes. Molecular Biology and Evolution, 28, 2359–2369. 21389272 10.1093/molbev/msr058

[mec14123-bib-0096] Yang, Z. , & Nielsen, R. (2000). Estimating synonymous and nonsynonymous substitution rates under realistic evolutionary models. Molecular Biology and Evolution, 17, 32–43. 10666704 10.1093/oxfordjournals.molbev.a026236

[mec14123-bib-0097] Zhang, X. (2008). The epigenetic landscape of plants. Science, 320, 489–492. 18436779 10.1126/science.1153996

[mec14123-bib-0098] Zhang, J. , Li, X. , He, Z. , Zhao, X. , Wang, Q. , Zhou, B. , … Liu, X. (2013). Molecular character of a phosphatase 2C (PP2C) gene relation to stress tolerance in *Arabidopsis thaliana* . Molecular Biology Reports, 40, 2633–2644. 23268310 10.1007/s11033-012-2350-0 PMC3563958

[mec14123-bib-0099] Zhang, Z. , Li, J. , & Yu, J. (2006). Computing Ka and Ks with a consideration of unequal transitional substitutions. BMC Evolutionary Biology, 6, 44. 16740169 10.1186/1471-2148-6-44 PMC1552089

[mec14123-bib-0100] Zhang, J. , Nielsen, R. , & Yang, Z. (2005). Evaluation of an improved branch‐site likelihood method for detecting positive selection at the molecular level. Molecular Biology and Evolution, 22, 2472–2479. 16107592 10.1093/molbev/msi237

[mec14123-bib-0101] Zheng, X. , Levine, D. , Shen, J. , Gogarten, S. M. , Laurie, C. , & Weir, B. S. (2012). A high‐performance computing toolset for relatedness and principal component analysis of SNP data. Bioinformatics, 28, 3326–3328. 23060615 10.1093/bioinformatics/bts606 PMC3519454

