# Supplementary material for: Adaptive sequence evolution is driven by biotic stress in a pair of orchid species (Dactylorhiza) with distinct ecological optima
Source: Mol Ecol. 2017 Apr 27;26(14):3649–62. doi: 10.1111/mec.14123 (PMC5518283; doi:10.1111/mec.14123)
Supplement: Supplementary file 1 [file MEC-26-3649-s001.pdf]

Supporting Information, including Supporting Tables and Supporting Figures

Adaptive sequence evolution is driven by biotic stress in a pair of orchid species (*Dactylorhiza*) with distinct ecological optima

Francisco Balao, Emiliano Trucchi, Thomas Wolfe, Bao-Hai Hao, Maria Teresa Lorenzo, Juliane Baar, Laura Sedman, Carolin Kosiol, Fabian Amman, Mark W. Chase, Mikael Hedrén, Ovidiu Paun

METHODS

RNAseq library construction and sequencing

The leaf material was fixed in RNAlater in the same morning, and was left at 4 °C overnight, before transferring it to -80 °C for storage until required. Total RNA was extracted using the RNeasy Plant Mini Kit (Qiagen) following the manufacturer's instructions. The purified RNA was stored at -80 °C. The concentration of RNA extracts was first measured with a NanoDrop ND-1000 Spectrophotometer (Thermo Scientific) and its purity estimated according to the wavelength ratio of A260/280. The quantification and quality of the RNA was then confirmed with an RNA 6000 Nano-kit on a 2100 BioAnalyzer (Agilent Technologies). The ribosomal RNA was depleted with the RiboMinus™ Plant Kit for RNA-Seq (Invitrogen) following the manufacturer's protocol. RNA fragmentation was done by hydrolysis for 2 min at 94 °C and used 2 µl 10x buffer RT (SuperScript III First-Strand Synthesis System for RT-PCR, Invitrogen) plus 75mM $MgCl_2$ in 12 µl concentrated RNA. After a cleaning step with an RNeasy spin column (from an RNeasy Plant Mini Kit, Qiagen), the first cDNA strand has been synthesized with the Superscript III First-Strand Synthesis System for RT-PCR (Invitrogen) and random hexamers. Surplus dNTPs have been eliminated with a Mini Quick Spin Column for DNA (Roche). The second strand cDNA synthesis has been performed with dUTPs, to allow for strand distinction upon Illumina sequencing. After a final clean up step with a MiniElute Reaction Cleanup kit (Qiagen) and quantification using a Quant-iT Picogreen dsDNA assay in a NanoDrop Fluorospectrometer ND-3300 (Thermo Scientific), the final RNAseq library preparation (using NEBnext Ultra RNA kit and an UGdase

treatment) and directional Illumina sequencing as 100bp paired-end reads was performed at the CSF Vienna (www.csf.ac.at/ngs). Samples fB4, iB0 and iS4 (Table 1) were sequenced as half lanes, whereas sample fP7 has been sequenced as two half lanes. Six other samples were sequenced as full lanes.

After controlling read quality with *FastQC* v0.11.2 (available from http://www.bioinformatics.babraham.ac.uk/projects/fastqc/), filtering of the raw reads was done with *Trimmomatic* v0.32 (Bolger et al., 2014) with default settings, except for increasing the threshold for average quality per base to 20 when scanning the reads over four-base sliding windows, and only retaining adaptor-free, high-quality reads that are at least 50 bases long. After quality filtering 78.6% of the read pairs were retained (Table S1). We further used only the paired-end filtered reads, as the single-end files produced by *Trimmomatic* contained only a negligible amount of orphan reads. The reads were finally filtered against a database of all whole genome Virus and Bacteria from NCBI (ftp://ftp.ncbi.nlm.nih.gov/genomes/Bacteria/ and ftp://ftp.ncbi.nlm.nih.gov/genomes/Viruses/ version 2014-07-03) by using *Bowtie2* v2.2.3 (Langmead and Salzberg, 2012) with default setting. Less than 1% of reads were found to be potential contamination from viruses or bacteria.

***De novo* transcriptome assemblies and annotation**

The transcriptome of each sequenced accession was individually assembled from its cleaned reads using *Trinity* version r20140413 (Grabherr et al., 2011; Haas et al., 2013) with a *kmer* setting of 25, strand-specificity (i.e., FR orientation) and retaining only contigs with a minimum length of 200 bp. One individual reference per species has been then retained based on the total length, N50 and the percentage of reads that uniquely mapped back to each reference by using the *CLC Genomics Workbench* v7.5 (Qiagen) with strand-specificity, a minimum similarity fraction of 0.95 over at least 0.95 of the length and mismatch/insertion/deletion costs of 2/3/3. A multi-individual assembly was also attempted for each species, however this failed to extend the N50 length significantly or increase the back-mapping rate (results not shown), and was discarded to avoid the risk of chimeric contigs.

Ecologically-relevant genes are expected to be generally expressed at low rates, and hence may be missed in individual assemblies. To account for this, we have retained for each accession the un-mapping reads when aligning them to the selected species-specific assembly, and these reads were further combined per species and were re-assembled with Trinity with

the same settings as above. The *D. fuchsii*, and, respectively, *D. incarnata* “lowly expressed” contigs were then added to their specific reference. A common reference for the two species has been constructed by pooling together the two individual references. We finally removed redundancy from each of the three constructed references by using the clustering algorithm of *CD-HIT-EST* (Fu et al., 2012) with a global identity of 80% over at least 70% of the length of the shorter sequence (i.e., -aS 0.7) and by comparing only the 5’-3’ strands (i.e., -r No). The final references are hereafter referred to as “f_reference”, “i_reference” and the *Dactylorhiza* (i.e., “f_i”) reference.

The individual Trinity assemblies contained between 7,686 (fA6, Table S1) and 70,193 contigs (iS8), with N50 ranging from 303 (for fA6) to 472 bp (for fB4). Based on the richness (i.e., the total length, Table S1), contiguity (i.e., N50 estimates) and completeness (i.e., mapping rate) we retained the assemblies of fB5 and iS8 as the best representative transcriptomes for *D. fuchsii* and respectively *D. incarnata*. In order to get better representation of the lowly expressed genes, species-specific Trinity assembly of all *D. fuchsii* and, respectively, *D. incarnata* reads that did not map to these reference transcriptomes were performed and resulted in 3,113 and 2,064 transcripts for *D. fuchsii* and, respectively, for *D. incarnata*. After combining the different assemblies and removing redundancy as explained above, the final combined fi_reference contains 33.8 Mbp within 101,010 transcripts (52.3% originated from *D. incarnata* and 47.7% from *D. fuchsii;* Table S2). This transcriptome will be of high value for further studies of gene expression alterations following whole genome duplication events in *Dactylorhiza*.

Functional annotation analyses of the fi_reference were performed in *Blast2Go* v.3.2.7 (Conesa and Götz, 2008) using cloud-based NCBI BLAST+ searches against the Viridiplantae database v. 17.01.2016 under the BLASTX algorithm and a minimum *e*-value of 10^{-6}. *Blast2Go* was further used to assign gene ontology (GO) terms to the contigs, to identify signatures of protein domains using InterProScan (Quevillon et al., 2005), and to annotate non-coding RNAs and cis-regulatory elements by performing Rfam scans against Xfam servers. Of the 101,010 transcripts in the fi_reference, 42.2% had significant BLASTX hits in Blast2Go. After mapping these contigs and integrating InterProScan results, 30,046 contigs were successfully annotated with GO terms (Fig. S1). In addition, 121 contigs received annotations from the Rfam scan, with 80 of them representing an rRNA type and 16 intronic regions.

Small RNA library preparation and sequencing

Isolation of RNA with smRNA enrichment was performed with the *mir*Vana miRNA Isolation Kit (Life Technologies) following the manufacturer's instructions. Up to 90 mg of the same tissue fixations as for the RNAseq experiment were used. The concentration of the raw smRNA isolates was measured with a Quant-iT Picogreen dsDNA assay (Invitrogen) and a NanoDrop Fluorospectrometer ND-3300 (Thermo Scientific). The quantification and quality of the RNA isolate was then confirmed with a 2100 Bioanalyzer (Agilent Technologies) using a small RNA analysis kit. The RNA extracts were denatured with loading buffer at 95°C for 3 min and further purified by gel size selection in a XCell *SureLock* Mini-Cell (Life Technologies) using a microRNA Marker (NEB) and 15% TBE-Urea pre-casted gels (Life Technologies) stained with 2x SYBR Green II (Life Technologies) for 1 h at RT. The smRNA samples were re-isolated from gel slices by overnight incubation in 0.3M NaCl at 4 °C, followed by filtration through Ultrafee-MC Durapore Filter Units 0.22µm (Merck Millipore) and precipitation for 2 h at -20 °C in 2.5x volume 100% ethanol, together with 1 µl GlycoBlue (Ambion). After resuspension in RNase-free water the smRNA isolates were again checked on a Bioanalyzer small RNA chip. For individual iB6 the isolate has yielded insufficient smRNA quantity and as no tissue was further available, the sample has not been processed further. The smRNA libraries were prepared with the NEBNext Multiplex Small RNA Library Prep Set for Illumina (Set 1 and 2, NEB) following the manufacturer's protocol, except for diluting the adapters 1:2 prior to use due to low sample concentration (130<mg) and using only 1.5 µl instead of 2.5µl of the primers. The samples were purified on a 1.5 % agarose gel with 1x TBE buffer. The DNA was extracted from the gel slices with a MinElute Gel Extraction clean-up Kit (Qiagen) and eluted in water. Illumina sequencing as a half-lane of 50 bp single-end reads was performed at the CSF Vienna (www.csf.ac.at/ngs). The individual samples were demultiplexed with the *BamIndexDecoder* tool of the *Illumina2Bam* software collection (available from http://gq1.github.io/illumina2bam/) and adapter sequences have been removed using *Trimmomatic* v0.30 (Bolger et al., 2014). After demultiplexing and adapters removal, the total number of reads longer than 15 nucleotides averaged ca. 9.9 million (std. 5.3 million) across all samples of the two species (*D. fuchsii* mean = 13.2, std. = 4.2; *D. incarnata* mean = 5.8, std. = 3.4).

References

Bolger AM, Lohse M, Usadel B. 2014. Trimmomatic: a flexible trimmer for Illumina sequence data. *Bioinformatics* **30**: 2114-2120.

Conesa A, Götz S. 2008. Blast2GO: a comprehensive suite for functional analysis in plant genomics. *Int. J. Plant Genomics* **2008**: 619832.

Fu L, Niu B, Zhu Z, Wu S, Li W. 2012. CD-HIT: accelerated for clustering the next-generation sequencing data. *Bioinformatics* **28**: 3150-3152.

Grabherr MG, Haas BJ, Yassour M, Levin JZ, Thompson DA, *et al.* 2011. Full-length transcriptome assembly from RNA-Seq data without a reference genome. *Nat. Biotechnol*. **29**: 644-652.

Haas BJ, Papanicolaou A, Yassour M, Grabherr M, Blood PD, *et al.* 2013. *De novo* transcript sequence reconstruction from RNA-seq using the Trinity platform for reference generation and analysis. *Nat. Protoc* **8**: 1494-1512.

Langmead B, Salzberg SL. (2012). Fast gapped-read alignment with Bowtie 2. *Nature Methods* **9**: 357-359.

Quevillon E, Silventoinen V, Pillai S, Harte N, Mulder N, Apweiler R, Lopez R. 2005. InterProScan: protein domains identifier. *Nucleic Acids Res*. **33**: W116-W120.

Table S1. Summary of individual RNAseq *Trinity* assemblies. The uniquely and, respectively, total mapping rates refer to mapping with *CLC Genomics Workbench* of the reads of one sample to the assembly produced by those respective reads with *Trinity*.

**Accession**	**Filtered pairs of reads (M)**	**Transcripts**	**Contigs**	**Total Megabases**	**N50 (bp)**	**GC (%)**	**Unique/Total mapping (%)**
fA6	183.9	7,686	6,163	2.5	303	50.3	-
fP1	40.5	15,373	13,348	5.9	391	51.0	85.4/85.7
fP7	112.5*	48,895	35,771	19.0	385	52.9	26.4/47.9
fB4	91.4	61,109	42,999	26.4	472	49.7	18.1/79.9
fB5	190.3	62,338	46,340	23.0	361	50.4	86.8/87.3
iA6	168.1	24,785	19,818	8.3	311	51.2	95.0/95.2
iS4	73.6	31,404	24,208	12.2	391	50.8	35.9/81.2
iS8	148.7	70,193	53,126	23.7	322	51.2	86.2/86.6
iB6	74.4	17,125	14,411	5.9	328	52.7	86.6/86.8
iB0	66.8	29,697	22,604	12.2	431	50.7	16.3/79.2

*summed over two half lanes

Table S2. Summary of the final reference transcriptomes.

Species	**Assembly**	**Transcripts**	**Contigs**	**Total base pairs**	**N50**
D. fuchsii	f_reference	54,596	43,834	19,420,738	356
D. incarnata	i_reference	61,841	49,960	20,394,752	314
Combined	fi_reference	101,010	88,461	33,794,033	319

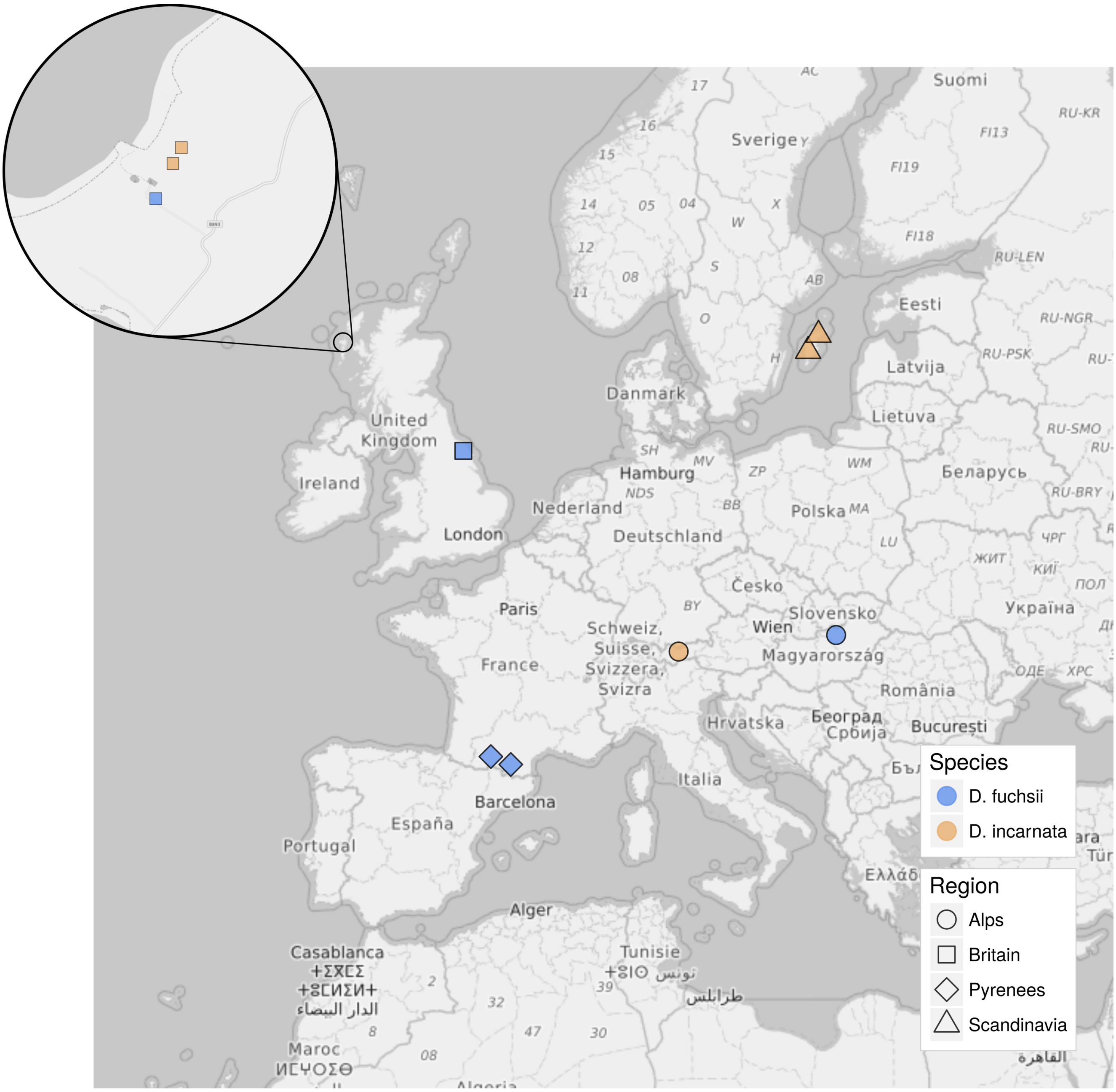

Supporting Figure S1. Map of the native localities of the *Dactylorhiza* samples analysed here. The samples were transplanted in a common garden at least one growing season before the material was fixed for analyses.

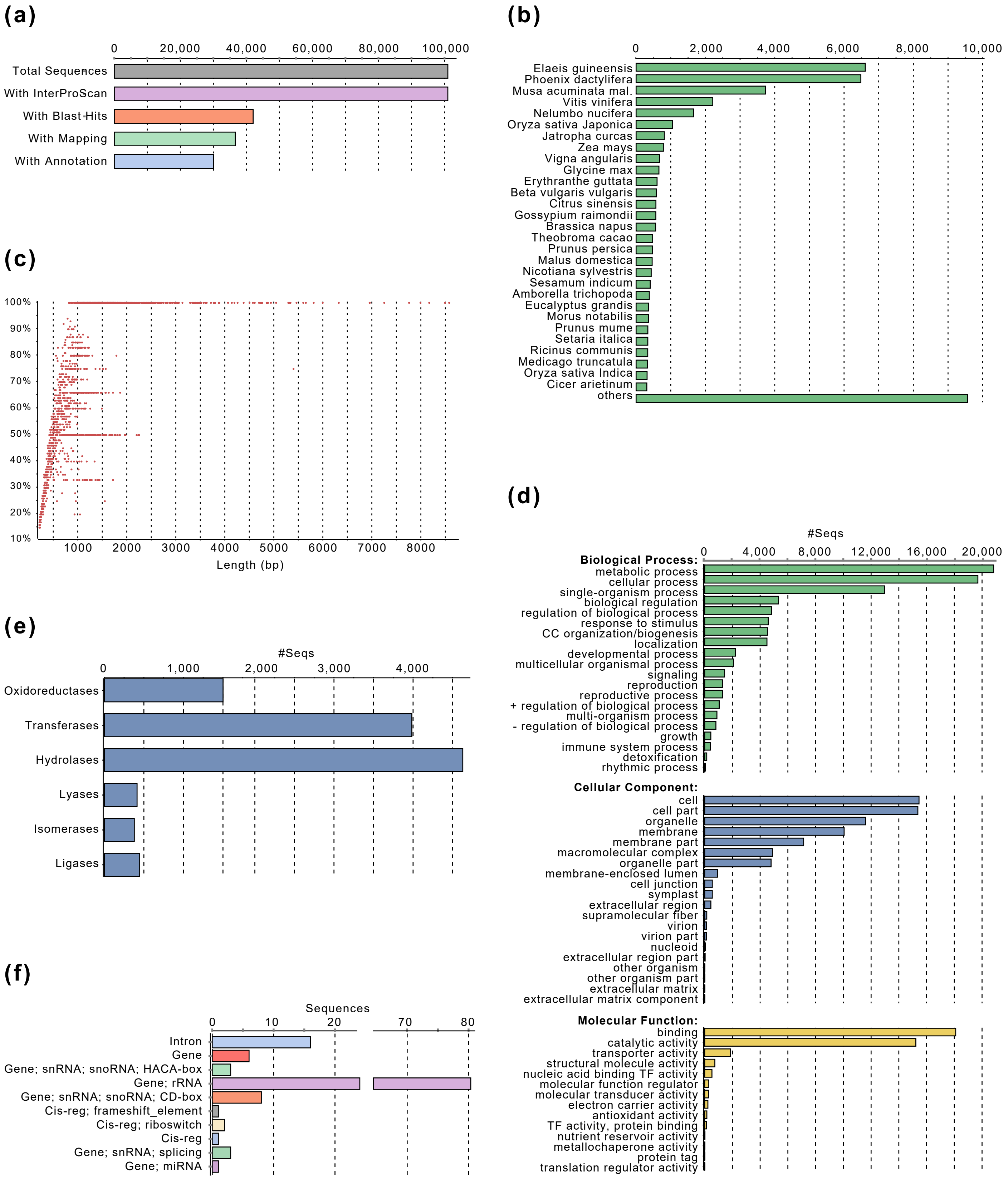

Supporting Figure S2. Results of annotation analyses of the combined *Dactylorhiza* fi_reference with Blast2Go. **(a)** Data distribution. **(b)** Blast top-hit species distribution. **(c)** Sequences with length(x) annotated. **(d)** GO distribution by level (2) - top 20. **(e)** Enzyme code distribution. **(f)** Rfam biotypes seqeunce distribution.

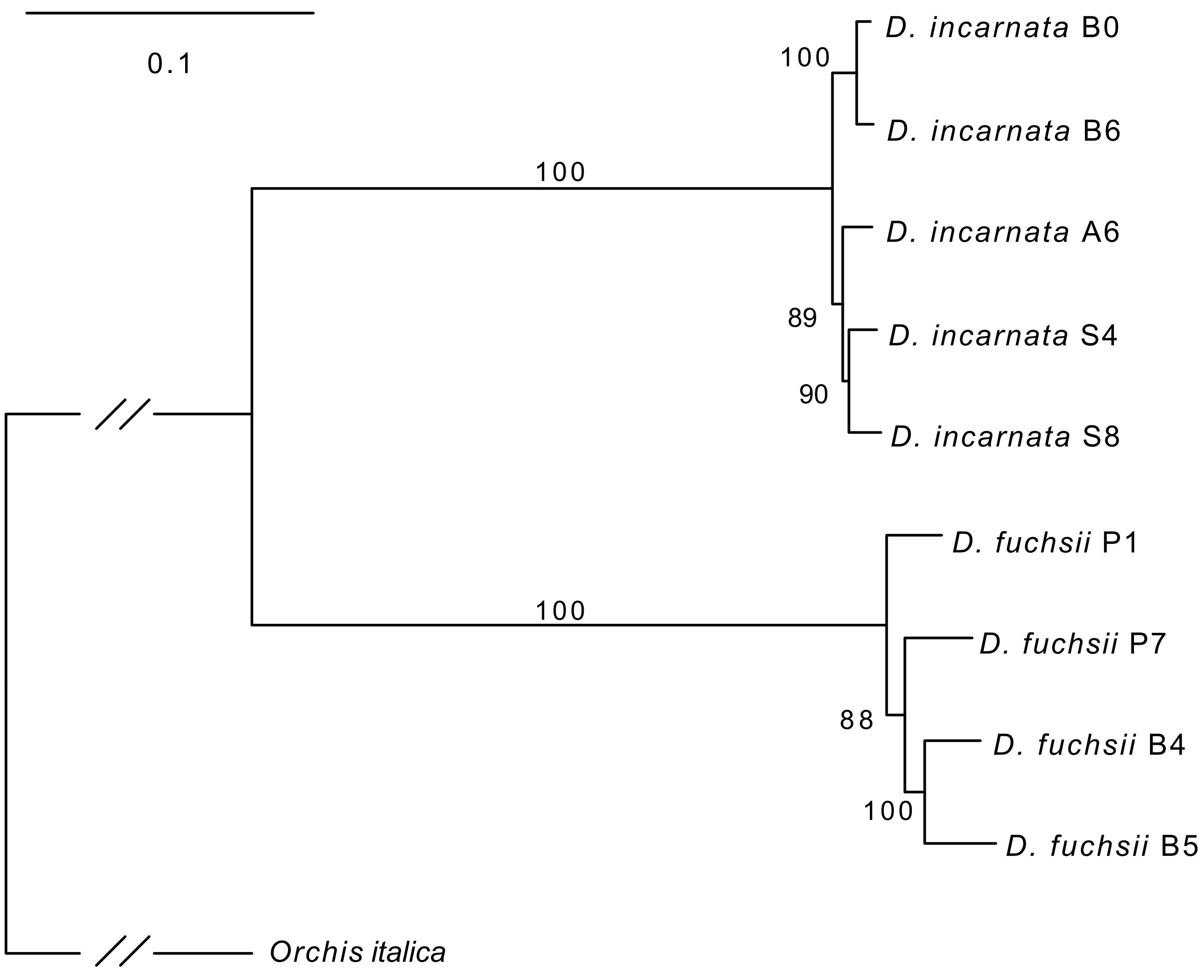

Supporting Figure S3. Maximum-Likelihood *RAxML* phylogenetic tree based on 449,518 high-quality cSNPs, illustrating relationships between the *Dactylorhiza* accessions analysed. Bootstrap percentages are indicated.

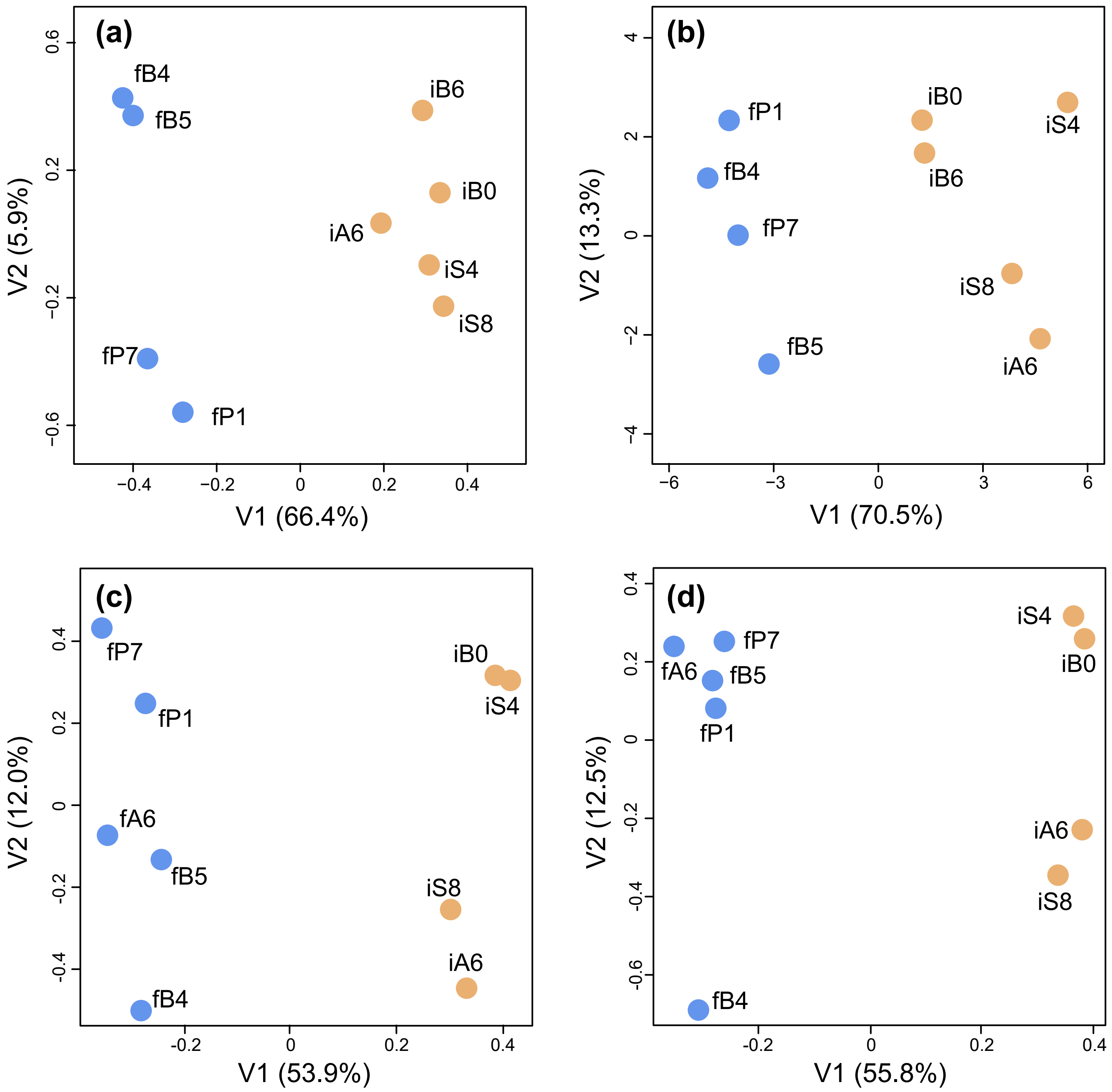

Supporting Figure S4. Transcriptome variation within and between *D. fuchsii* and *D. incarnata*. **(a)** *SNPRelate* PCA on 129,511 filtered biallelic cSNP variants. **(b)** PCA showing the largest components of variance in gene expression as uncovered by *edgeR*. **(c)** PCA drawn with *EDAseq* on the patterns of expressed miRNA and tasiRNA (i.e., analysis of 20-22nt small RNAs). **(d)** *EDAseq* PCA on patterns of siRNA expression (i.e., analysis of 24nt small RNAs).

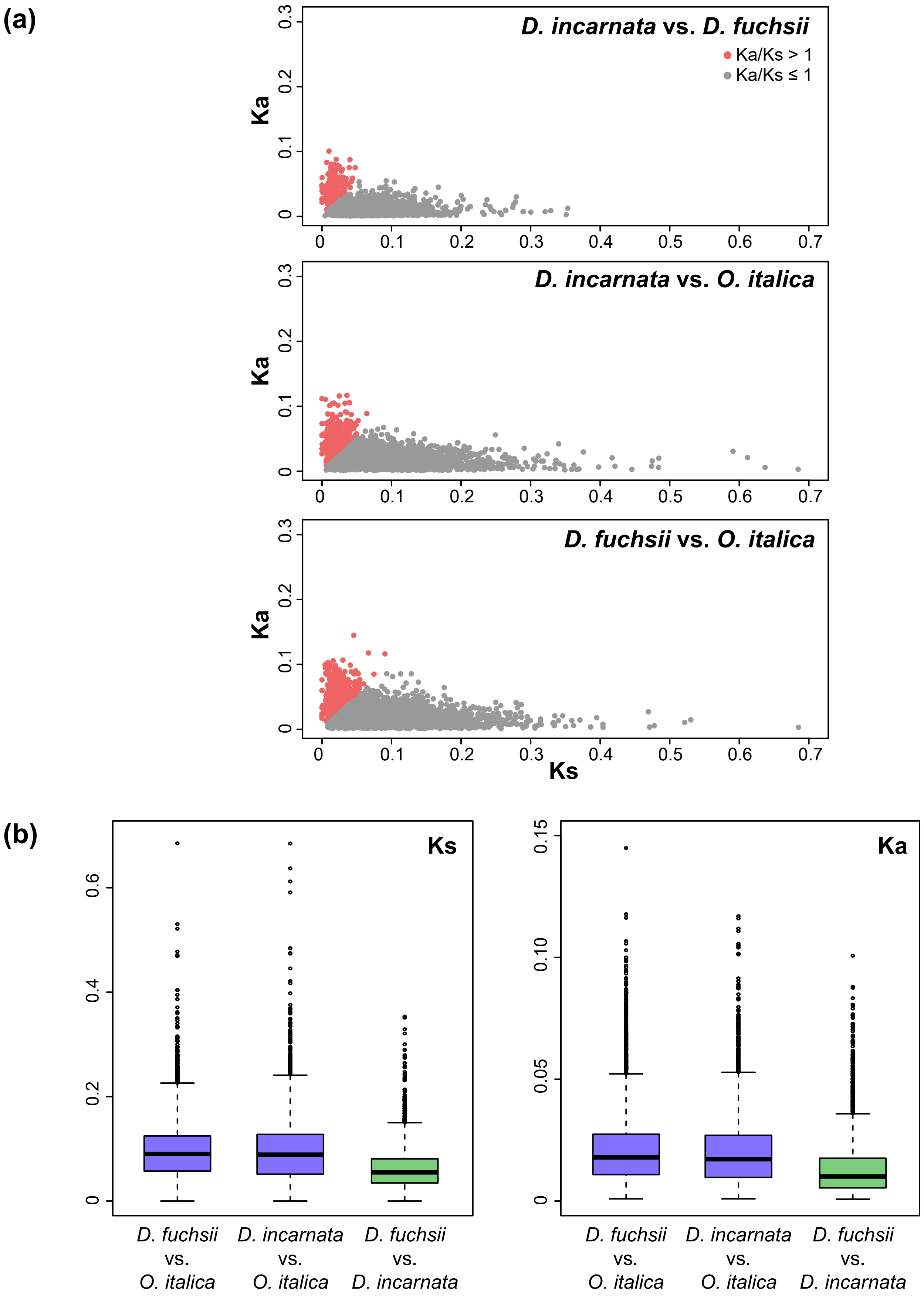

Supporting Figure S5. Representation of the synonymous and non-synonimous sustitution rates. **(a)** KaKs plots of pairwise analyses. The red dots represent the putative CDS showing signals of positive selction; the grey dots indicate putative CDS that show signals of purifying selction. **(b)** Boxplots of the synonymous substitution rates (Ks) and the non-synonymous substitution rate (Ka) in intergeneric (blue) and intrageneric (green) comparisons. Note the different scales on the Y-axes.

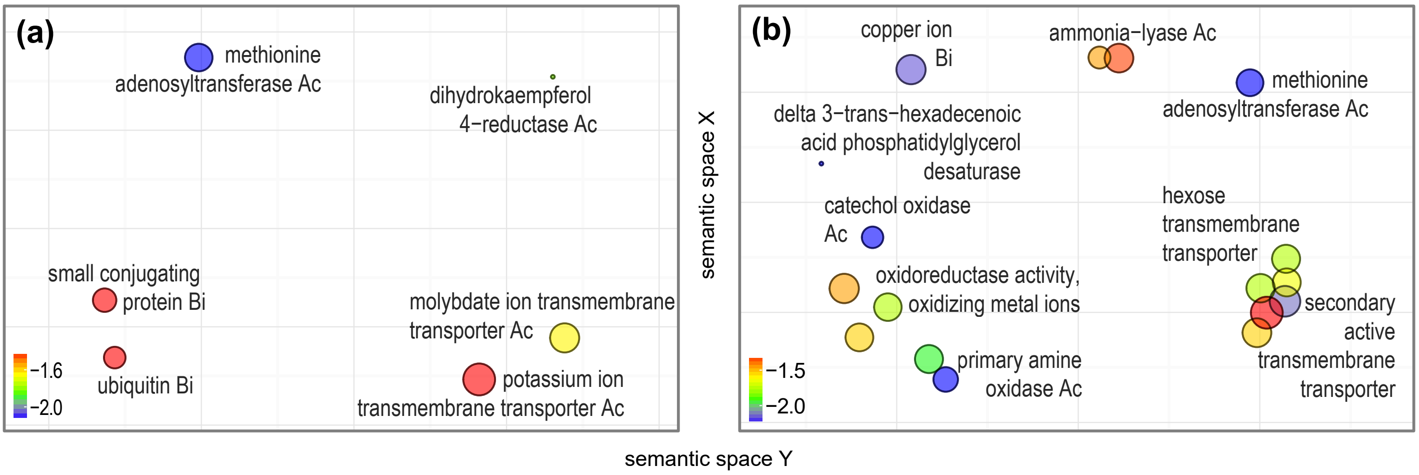

Supporting Figure S6. Enriched molecular functions ($p < 0.01$) with elements targeted by positive selection in *D. fuchsii* (**a**), and in *D. incarnata* (**b**). Bubble size is proportional to the frequency of the respective term in the public GO database. The colour represents the log10 value of the significance of the Fisher's tests of enrichment, corresponding to the indicated scale. Ac - activity; Bi - binding.

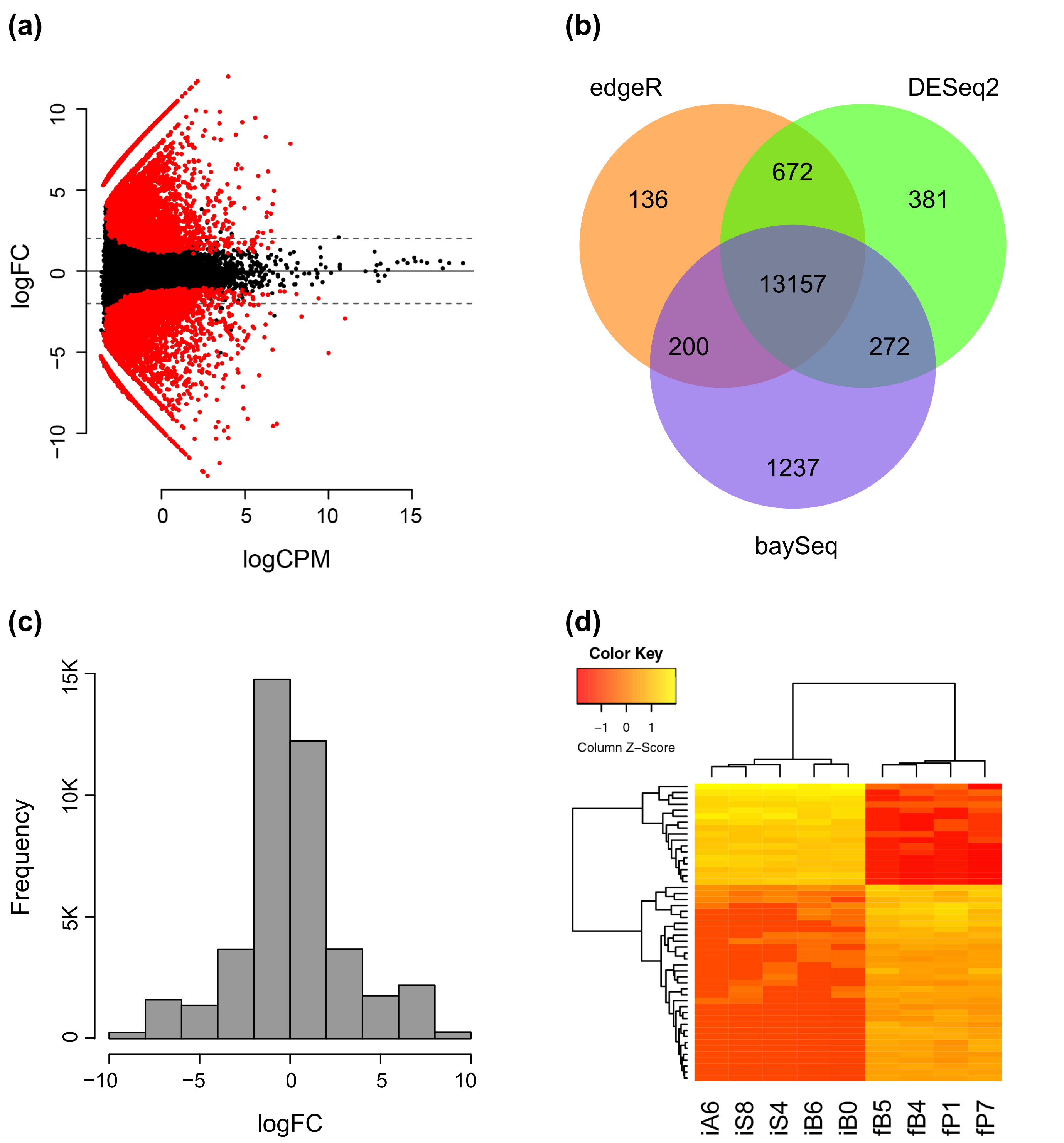

Supporting Figure S7. Differential gene expression analysis of *D. incarnata* versus *D. fuchsii*. **(a)** edgeR drawn MA-plot of the relative expression levels. The x-axis shows the log2 of the counts per million of mapped reads (CPM) for each cluster. The y-axis shows the log2 of the expression fold change (FC) for each transcript. The red dots represent the clusters that were DE between the two *Dactylorhiza* species. **(b)** Intersection of the DE results of the three tests performed at a level of false discovery rate (FDR) of 0.05. **(c)** Histogram of the log2 of the expression fold change values. **(d)** Heat map of the top 50 most differentially expressed clusters between *D. incarnata* and *D. fuchsii*.

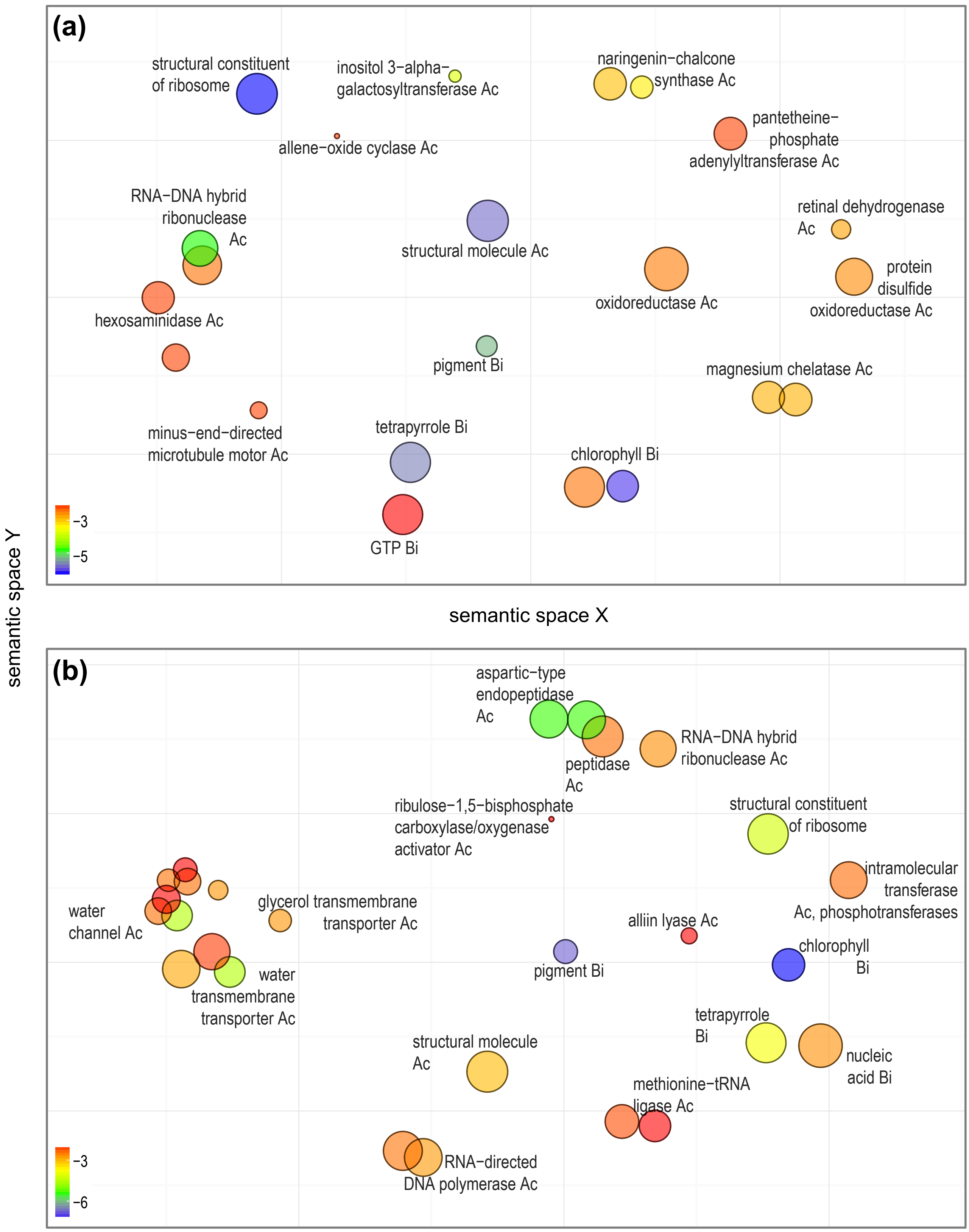

Supporting Figure S8. Enriched molecular functions ($p < 0.01$) that are affected by overexpression in *D. fuchsii* (**a**) and *D. incarnata* (**b**). The log10 of the *p*-value of the enrichment test is shown by the colour of the bubbles, according to the indicated scale. The size of the bubbles is proportional with the frequency of that particular GO term in the public GO database. Ac, activity; Bi, binding.

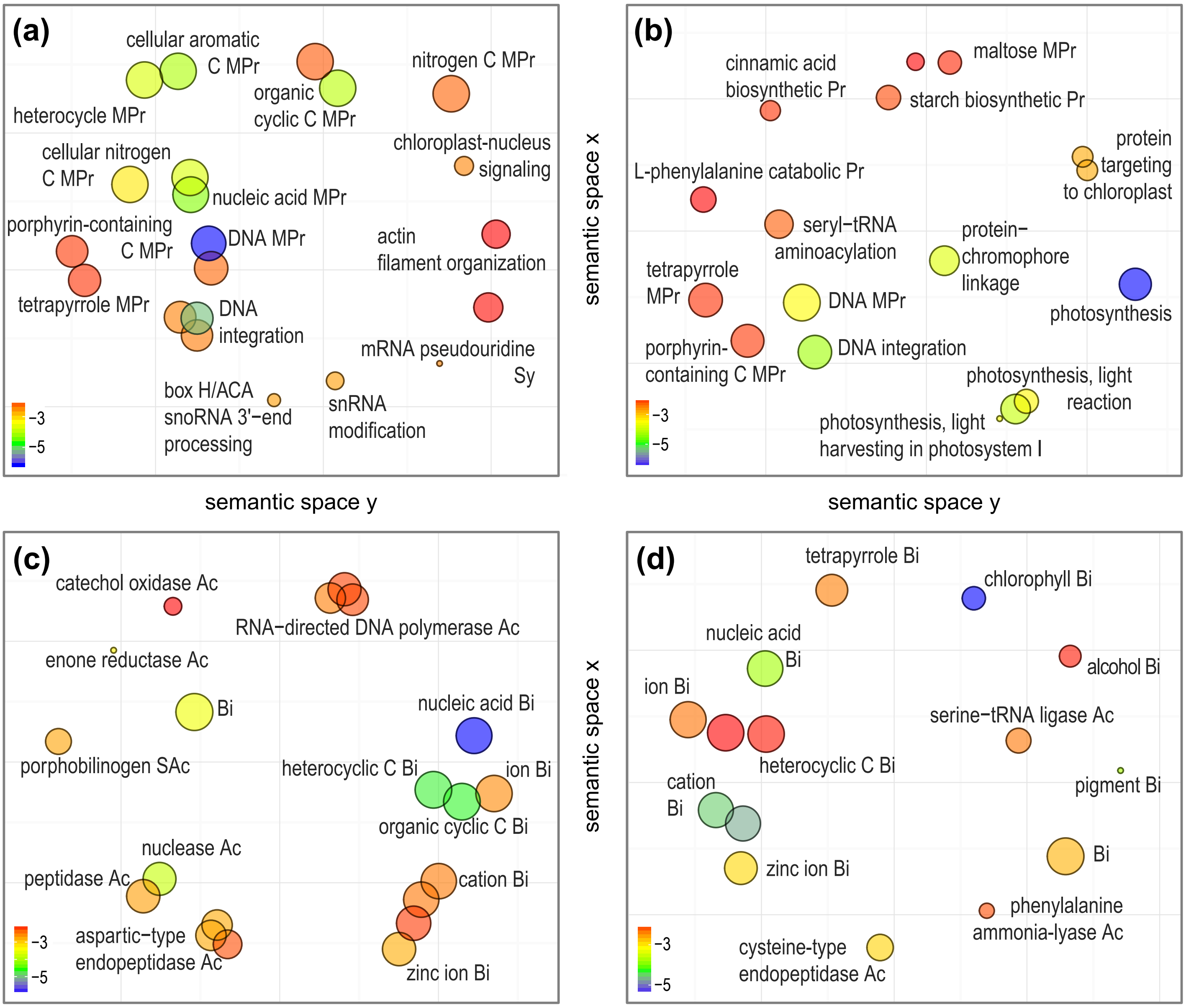

Supporting Figure S9. Enriched (p < 0.01) biological processes (**a-b**) and molecular functions (**c-d**) with elements differentially targeted (FDR < 0.05) by miRNAs/tasiRNAs between *D. fuchsii* and *D. incarnata*. **(a, c)** Enriched GO terms of genes with increased mi/tasiRNA targeting in *D. fuchsii*. **(b, d)** Enriched GO terms of genes with over-regulation by mi/tasiRNAs in *D. incarnata.* Bubble size is proportional to the frequency of the respective term in the public GO database. The colour represents the log10 value of the significance of the Fisher's test of enrichment, corresponding to the indicated scale. C, compound; Ac, activity; SAc, synthase activity; Bi, binding; Sy, synthesis; Pr, process; MPr, metabolic process.

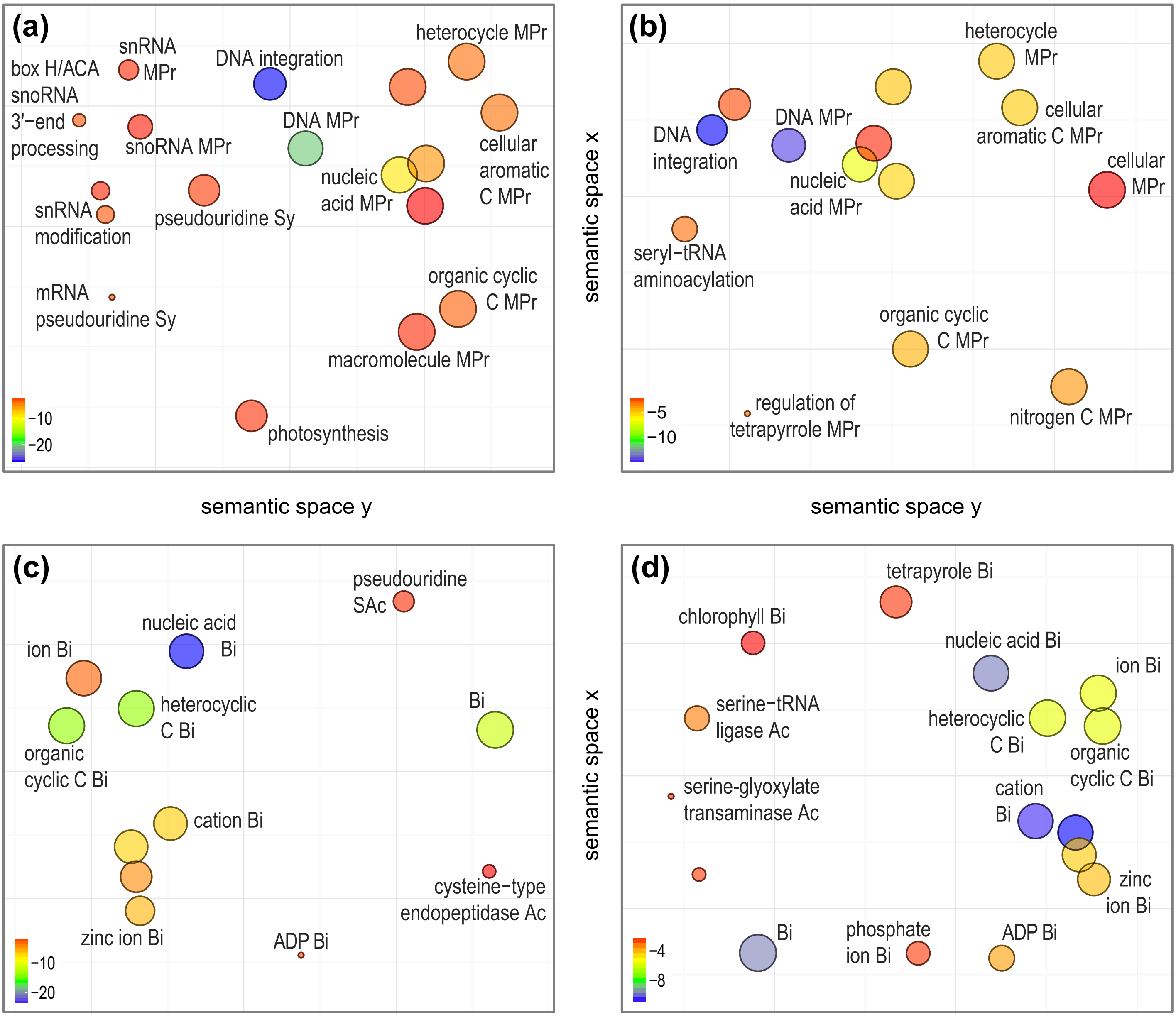

Supporting Fig. S10 Enriched (*p* < 0.01) biological processes (**a-b**) and molecular functions (**c-d**) with elements differentially targeted (FDR < 0.05) by siRNAs between *D. fuchsii* and *D. incarnata*. **(a, c)** Enriched GO terms of genes with increased siRNA targeting in *D. fuchsii*. **(b, d)** Enriched GO terms of genes with over-regulation by siRNAs in *D. incarnata.* Bubble size is proportional to the frequency of the respective term in the public GO database. The colour represents the log10 value of the significance of the Fisher's tests of enrichment, corresponding to the indicated scale. C, compound; Ac, activity; SAc, synthase activity; Bi, binding; Sy, synthesis; Pr, process; MPr, metabolic process.
